# Supplementary material for: A changing thermal regime revealed from shallow to deep basalt source melting in the Moon
Source: Nat Commun. 2022 Dec 9;13:7594. doi: 10.1038/s41467-022-35260-y (PMC9734159; doi:10.1038/s41467-022-35260-y)
Supplement: Supplementary file 1 — Supplementary Information for [file 41467_2022_35260_MOESM1_ESM.pdf]

Supplementary Information for

A changing thermal regime revealed from shallow to deep basalt source melting in the Moon

Yash Srivastava[1,2], Amit Basu Sarbadhikari[1,*], James M. D. Day[3], Akira Yamaguchi[4], Atsushi Takenouchi[4,5]

[1]Physical Research Laboratory, Ahmedabad 380009, India

[2]Indian Institute of Technology Gandhinagar, Gujarat 382355, India

[3]Scripps Institution of Oceanography, University of California San Diego, La Jolla, CA 92093-0244, USA

[4]National Institute of Polar Research (NIPR), 10-3 Midori-cho, Tachikawa, Tokyo 190-8518, Japan

[5]The Kyoto University Museum, Kyoto University, Yoshida-honnmachi, Sakyo-ku, Kyoto-shi, Kyoto, 606-8501, Japan

E-mail: amitbs@prl.res.in

Contents of this file

Supplementary Text 1 to 8
Supplementary Figures 1 to 14
Supplementary Table 1
Supplementary References

Supplementary Text 1. Petrography and mineral chemistry of Asuka-881757

The lunar meteorite A-881757 is a coarse holocrystalline, unbrecciated gabbro. The meteorite comprises large grains of pyroxenes (2-8 mm) and plagioclase (1-3 mm) along with relatively small (< 1 mm) discrete patches occupied by late-stage crystallizing phases (symplectites and mesostasis) (Supplementary Fig. 1). Symplectites are characterized by vermicular intergrown olivine, pyroxene, and silica assemblage. The mesostasis phases include fayalite, troilite (FeS), ilmenite ($FeTiO_3$), silica (tridymite?), a P-rich phase, K-feldspar, and spinel.

Pyroxene ranges in a broad spectrum of compositions, with increasing iron contents from relatively Fe-poor cores ($Wo_{14-32}Fs_{31-43}$) to Fe-rich ($Wo_{20-35}Fs_{52-63}$) rims (Supplementary Data 1 and Supplementary Fig. 3a). The variation of Al/Ti with Mg# indicates that the core of pyroxene has relatively consistent Al/Ti (~3); the ratio gradually decreases from 3 to ~1.5 as plagioclase appears (Supplementary Fig. 3b). Near some pyroxene rims, the Al/Ti ratio becomes consistently ~1, signifying the occurrence of Fe-Ti oxides in the crystallization sequence. The pyroxenes show very fine exsolution features (~1 μm) visible under a scanning electron microscope at 10 μm resolution (Supplementary Fig 2a).

Plagioclase grains have retained their relict compositional igneous zoning even after maskelynitization. The zoning in maskelynitized plagioclase grains ranges from An_{96} cores to An_{87} rims (Supplementary Fig. 4). However, in close contact with late crystallizing phases, rims are An_{75} (Supplementary Fig. 4).

Symplectites and mesostasis occur throughout the sections and are usually associated with the Fe-rich rims of pyroxene. Fayalite, Fe-rich pyroxene, and silica are the main constituents of symplectites. The mesostasis phase shows a close relation to the wide fractures. Fayalitic olivine (Fo_{8-10}) and silica dominate the mesostasis phases. The olivine grains (Fo_{8-10})

are fayalitic (Fe-rich) compared to most of the returned mare basalt samples. The silica phase in the mesostasis occurs either as elongated laths (<2 mm) or as small anhedral (<200 μm) aggregate grains (Supplementary Fig. 2b). Spinel infrequently occurs as irregular, interstitial grains (<200 μm). Spinels are rich in Cr and commonly intergrown with ilmenite in the mesostasis and are primarily associated with fayalitic olivine (Fa) rim of 20-90 μm thickness (Supplementary Fig. 2c). Spinel shows a compositional range within chromite-ulvöspinel solid solution ($2Ti_{52-85}$, Al_{6-15}, Cr_{7-32}) (Supplementary Data 1). Troilites (FeS) are aggregates of subrounded grains with sizes ranging from tiny 20 μm grains (present within the pyroxene rims) to relatively coarse ~300 μm grains. Apatites of ~200 μm size also share boundaries with troilite grains (Supplementary Fig. 2d). Ilmenite has almost no variation among the textural varieties (Supplementary Data 1). A highly vesicular fusion crust occurs in the sections studied and has a similar chemical composition to the whole rock (Supplementary Data 1).

Supplementary Text 2. Other lunar meteorites

YAMM basalts

Like sample A-881757, other YAMM meteorites are unbrecciated except MET 01210, a regolith breccia with basaltic clasts (~70 vol. %; ref. [1]). A-881757 and MIL 05035 show identical coarse-grained textures with grains ranging from a few to several millimeters[2-7]. Yamato-793169 and MET-01210 also contain materials with coarse-grained textures but with smaller average grain sizes. The pyroxene (Supplementary Fig. 3a), plagioclase (Supplementary Fig. 4; An_{77-96}), fayalitic olivine, and chromian ulvospinels show identical compositional range among the group. The pyroxenes of YAMM have similar Ti/Al and Mg# vs. Ti# (Ti/Ti+Cr) trends (Supplementary Fig. 3b), suggesting a similar crystallization path with a common parent magma composition [2]. YAMM meteorites are all low-Ti basalts with

common crystallization age of ~3.9-3.8 Ga[8-10]. Further, the rare earth element (REE) patterns are similar for MIL 05035, Y-793169, and A-881757 (Fig. 3).

Kalahari 009

Kalahari 009 is a monomict breccia with basaltic mineralogy and composition (Supplementary Data 2; ref. [11]). It is the oldest mare basalt with an age of 4.35 Ga[12]. The basaltic clast has a coarse-grained sub-ophitic texture with grains up to 4 mm. Pyroxene and plagioclase are the primary constituents of the rock, while olivine and other phases are accessories. Like A-881757, olivine and pyroxene in Kalahari 009 are Fe-rich, and silica occurs in symplectite. The pyroxene and plagioclase show a compositional range of $Wo_{6-41}Fs_{22-67}$ and An_{95-72}, respectively (ref. [11]). The meteorite is a VLT basalt with unusually low REE abundance (Fig. 3 and Supplementary Data 2).

Luna 24 ferrobasalts

Luna 24 ferrobasalts are soil fragments with ophitic pyroxene and olivine phenocrysts. Other accessory phases are spinels, ilmenite, feldspar, and silica. Pyroxene composition varies from Ca-poor ($Wo_{12}Fs_{36}$) to Ca-rich ($Wo_{25}Fs_{50}$). Olivine is zoned from Fo_{60} to Fo_5 (ref. [13]). Plagioclase shows compositional variation from An_{96} to An_{86} (ref. [13]). The soil fragments are low-Ti and marginally high–Al and low-K. The REE abundance of these basaltic rock fragments is low and shows LREE depletion (Fig. 3 and Supplementary Data 2), similar to sample A-881757 (ref. [14]).

NWA 032

NWA 032 is a low-Ti unbrecciated basalt with subophitic-ophitic magmatic textures. The phenocryst population is composed predominantly of olivine (Fo_{65-60}), pyroxene ($Wo_{20-40}Fs_{50-}$

$_{60}$ and $Wo_{10-20}Fs_{75-85}$ domains), and a small amount of chromite along with a groundmass of feldspar (~An_{85}), pyroxene (Wo_{15-25} Fs_{75-99}), ilmenite, troilite, and trace metals[15]. The REE concentrations yield a well-defined Eu anomaly (Fig. 3), and an LREE enriched pattern[15].

LAP basalts

Low-Ti lunar mare basalt meteorites consist of LAP 02-205, -224, -226, -436, and LAP 03-632, collectively known as LAP basalts. They are holocrystalline basalts containing similar proportions of pyroxene, plagioclase (An_{79-93}), ilmenite, troilite, and free silica, in addition to variable quantities of K-rich glass, ulvöspinel, chromite, phosphates, Fe-Ni metals, baddeleyite, , fayalitic (<Fo_{20}) and relatively Mg-rich (Fo_{20-67}) olivine[1]. Pyroxenes show comparable variation from the Mg-rich cores to extremely Fe-rich rims: $Wo_{9-41}Fs_{21-90}$ (LAP 02205), Wo_{8-39} Fs_{23-86} (LAP 02224) and $Wo_{8-40}Fs_{22-87}$ (LAP 02226) (ref. [16]). Plagioclase composition varies from: An_{91-81} (LAP 02205), An_{90-84} (LAP 02224) and An_{90-84} (LAP 02226) (ref. [16]). These meteorites are low-Ti, low-Al, and low-K basalts (Fig. 2 and Supplementary Data 2). They show elevated REE abundances relative to the other low-Ti basalts (Fig. 3; ref. [17]).

Supplementary Text 3. Estimation of parental melt and source composition

To understand the source conditions, the studied samples must represent a parental melt in chemical equilibrium with the mantle source. Earlier studies based on isotopes including Sm-Nd, Rb-Sr, U-Pb, and Lu-Hf, have highlighted the depleted mantle source characteristics of YAMM and other non-KREEP basalts[4,8,10,11,18,19] (Supplementary Fig. 8). Since the studied rocks are Fe-rich (Mg# 33-49), estimation of their parent melts is challenging. The low Mg#

can either account for the result of extensive fractional crystallization[1,20,21] or a product of (low-degree) partial melting of heterogeneous Fe-rich mantle[18,19,22] (Supplementary Fig. 10). To address this problem, we examined whether the rocks represent the original melt composition or show any signs of modification via processes such as assimilation and/or crystal fractionation. Our approach was first to identify if there is any KREEP component in these rocks by considering the rare earth element (REE) composition. Then, we tested any assimilation effect in the studied rocks by considering REE in the most abundant silicate phase pyroxene and plotting the rocks in isotopic ratio diagrams. Finally, we assessed whether the rocks went through extensive fractionation processes to yield their high Fe-content bulk by considering the exchange coefficients of Fe-Mg between the melt and the most Mg-rich silicate phases that crystallized first from the melt.

Previous studies have shown that changes in the parental melt composition can be traced from their REE, as the distinct source characteristics and magmatic processes would significantly affect the REE composition of parent melt[17,23]. The absence of characteristically high ratios of HFSE/LREE (HFSE: high field strength element) and LREE/HREE of urKREEP in the studied rocks (A-881757 and other YAMM meteorites) suggest they are devoid of the KREEP component (ref. [18,24]; this study). Also, using the approach of Day et al[17], we track the assimilation effect, if any, through the evolving REE composition of pyroxene. We find a systematic variation of REE in the pyroxenes of MIL 05035 (La/Yb= 0.06-0.17, ref. [4,5]) and of the LAP basalts (La/Yb = 0.14-0.26, ref. [17]), which is consistent with the fractionation trend further implying that no assimilation has occurred in these rocks. Lastly, a plot for the $^{87}Rb/^{86}Sr$ source region versus $^{147}Sm/^{144}Nd$ source region provides additional evidence for these rocks' unassimilated source (Supplementary Fig. 8).

Next, we assess whether the whole-rock composition of the samples is in equilibrium with the observed mineral (core) composition, using the exchange coefficients, $K_{D,Fe-Mg}^{Ol-melt}$ =

0.32 ± 0.03 (ref. [25]) and $K_{D,Fe-Mg}^{Pyx-melt}$ = 0.28 ± 0.03 (ref. [26]). YAMM, Kalahari 009 and Luna 24 show signs of partial mineral (olivine and pyroxene) accumulation while olivine and pyroxene composition of NWA 032 and LAP basalts are in-equilibrium with the whole rock (Supplementary Fig. 7). The accumulation of minerals restricts these samples to be fractionated products.

As the measured whole-rock composition most likely reflects an original melt composition, we discuss the parental melt composition of each rock in the following sub-sections. Later, using an appropriate parental melt composition, we deduce a probable source composition of each studied rock by performing REE modeling under the backdrop of the lunar magma ocean (LMO) hypothesis (Methods; ref. [18,23,27,28]). This REE modeling initially assumes that the LMO has a source composition of 3 x CI for the REE[29] and then calculates the amount of partial melting required to produce each basalt group from the best fit source composition (Methods; ref. [23,27]). As YAMM, Kalahari, and Luna 24 ferrobasalts show a slight negative to slight positive Eu-anomalies, the best fit mineralogy was obtained close to 75-80 % LMO crystallization (percent solid) (Supplementary Figs. 9 and 14), because plagioclase reaches a liquid phase at this point[7,30,31]. Northwest Africa 032 and LAP basalts show a high REE abundance (~ 40-50×CI abundance of LREE and ~30-43×CI abundance of HREE) with a prominent negative Eu-anomaly, suggesting their source must have formed after the plagioclase separation (much after 78 % crystallization) during the LMO crystallization (Fig. 3). Our estimated best fit mineralogy for NWA 032 and LAP basalts was obtained at 86 % crystallized solid (PCS) with ~1 % TIRL (Supplementary Figs. 9 and 14). The trace element modeling results of each sample are discussed in the following sub-sections.

YAMM basalts

A-881757 and MIL 05035 both show a similar bulk major-element composition. The MELTS model was run at oxygen fugacity (IW-1) relevant to the Moon. For the bulk composition of A-881757 (Mg# 38.3), the first appearing silicate phase is the pyroxene of Mg# 69. However, the most magnesian pyroxene in our studied A-881757 sections is Mg# 54, likely suggests a possible cumulate component within the sample. Our analysis of the bulk composition of MIL 05035 also shows a similar result. The presence of the cumulate component is also favored by the coarse-grained texture as well as the lower abundance of incompatible trace elements (~6×CI abundance of LREE and ~18×CI HREE) than in Apollo mare basalts (> ~60×CI for LREE and >~40×CI HREE) (Fig. 3). Moreover, the La/Sm versus La and Ce/Yb versus Yb plots (Supplementary Figs. 5 and 6) highlight partial melting relations in the clan members rather than fractional crystallization trends. Based on its lowest ITE abundance within the clan, we chose MIL 05035 as the prime member to have a composition in equilibrium with the parental melt. The measured low-ITE and low Mg# of YAMM basalts suggest that the modal mineralogy of the mantle forming these basalts must be low in an early formed olivine component and be comprised of later crystallized minerals such as orthopyroxene and low-Ca pyroxenes (high clinopyroxene/olivine ratio). The retention of a limited quantity of plagioclase is also required to produce the observed small negative Eu anomalies in these samples. The deduced modal mineral abundance (17 vol.% olivine + 23 vol.% orthopyroxene + 59 vol.% pigeonite + 1 vol.% plagioclase) for the YAMM's parental melt fits best with a source formed at 75-80 % LMO crystallization (75-80 PCS). Similar modeled mineralogy at 75-80 PCS have been suggested by experimental and thermodynamical models of LMO crystallization[30,31]. Our trace element modeling shows that the source of these basalts underwent approximately 3-6% partial melting of the mantle with the composition of 75-80 PCS + 1% TIRL (Supplementary Data 3, Supplementary Figs. 9 and 14), which supports earlier analytical and experimental observations[2,4,5].

Kalahari 009

The maximum Mg-rich olivine and clinopyroxene observed in Kalahari 009 is Fo_{48-50} and Mg# ~70, respectively (ref. [11]). Our calculated olivine composition (Fo_{74}) is not present in the sample, in equilibrium with the whole rock. Instead, the observed pyroxene of Mg#70 suggests conditions close to equilibrium. There can be two possibilities. The most magnesian clinopyroxene is in equilibrium with the parental melt. Otherwise, Kalahari 009 got fractionated from a more primitive parent magma than its bulk. The difference in the whole rock (Mg# 49) and the observed mineral composition suggests the accumulation of more mafic minerals. It restricts them from being the product of extensive fractional crystallization (Supplementary Fig. 7). Furthermore, extreme fractional crystallization seems least likely, as Kalahari 009 contains unusually low ITE (~ 3×CI abundance of LREE and ~8×CI abundance of HREE) (Fig. 3). For these reasons, we assume that the whole rock composition of Kalahari 009 closely approximates the parental melt composition, although it may contain a cumulate component.

The relatively high Mg# (~49) of Kalahari 009 compared with other KREEP-free basalts indicates that the clinopyroxene (pigeonite)/olivine ratio in the mantle source of Kalahari 009 must be lowest among the studied KREEP-free basalts. The REE composition of Kalahari has a slight positive Eu anomaly suggesting that the source might have formed after plagioclase appeared on the liquidus (i.e., after 75 PCS) and the observed positive Eu anomaly is resultant of retention of plagioclase in the mantle source due to incomplete plagioclase separation[27]. The deduced modal mineral abundance of Kalahari 009 source at 75-80 PCS is 30 vol.% olivine + 23 vol.% orthopyroxene + 40 vol.% pigeonite + 7 vol.% plagioclase. The parental melt composition of Kalahari 009 indicates approximately 7-9% partial melting of early cumulate (75-80 PCS + 1% TIRL) (Supplementary Fig. 9).

Luna 24 ferrobasalts

Luna 24 shows a similar compositional and mineralogical trend to YAMM basalts and other studied samples. They have low Mg# (35.7) with primary olivine (~Fo_{58}) as well as pyroxene core (Mg# ~60) which are somewhat in equilibrium with the bulk rock (Supplementary Fig. 7). Assuming a bulk rock with a low ITE concentration close to a parental composition (Fig. 3), the trace element modeling implies approximately 3-6% partial melting of the source at 75-80 PCS + 1 % TIRL. The low Mg and low-ITE in the Luna 24 ferrobasalts suggest that the mantle source of these basalts is rich in clinopyroxenes (pigeonite and small amount of augite). The best fit source mineralogy obtained for Luna 24 ferrobasalts at 75-80 PCS contains 25 vol.% olivine, 23 vol.% orthopyroxene, 48 vol.% pigeonite, 2 vol.% augite and 2 vol.% plagioclase (Supplementary Fig. 9). The modelled mantle mineralogy shows minor plagioclase retention which is consistent with the result from previous studies[22,32] and is likely because of Al-rich nature of these rocks. Ma et al.[14], based on observation of varying REE coupled with nearly constant Eu and Sc, also arrived at similar conclusion that small amount of clinopyroxene and plagioclase retained in the mantle source of Luna 24 ferrobasalts.

NWA 032 and LAP basalts

Whole-rock Mg# for the NWA 032 (38-40; ref. [15,33]) and the LaPaz basalts (34–38; ref. [17]) suggest that they are in equilibrium with their primary crystallizing mineral phase, i.e., olivine cores, Fo_{60-65} and $Fo_{58–64}$, respectively. Unlike previous samples, NWA 032 and LAP basalts show an elevated ITE abundance (Fig. 3 and Supplementary Data 2). However, based on Sm-Nd and Rb-Sr isotopic studies[18,19], it has been suggested that the elevated abundance of these samples is not related to KREEP enrichment but rather a low degree of partial melting of Fe-rich mantle (Fig. 2). Following this logic, we assume that the whole rock composition closely

approximates the parental melt composition of both NWA 032 and LAP basalts. Despite the isotopic difference between NWA 032 and LAP basalts (Fig. 2), the modal mineralogy of their sources at 86 PCS is somewhat similar. The 86 PCS source has previously been suggested to be mantle source of many Apollo mare basalts, however, those mare basalts are Mg-rich compared to NWA 032 and LAP basalts. This is indicative that the mantle source of these basalt contains later crystallized Fe-rich components of the LMO, with high abundance of clinopyroxenes (pigeonite and augite) relative to early formed olivine and orthopyroxenes. The modeled source comprises 10 vol.% olivine + 23 vol.% orthopyroxene + 46 vol.% pigeonite + 21 vol.% augite for NWA 032 and 10 vol.% olivine + 23 vol.% orthopyroxene + 46 vol.% pigeonite + 20-21 vol.% augite + ≤ 1 vol.% plagioclase for LAP basalts. The results obtained for these samples suggest a very low degree (~0.7-1.5 %) of partial melting in the 86 PCS + 2% TIRL source can generate the REE abundance of NWA 032 and LAP basalts (Supplementary Fig. 9), similar to the results of Borg et al.[18].

Supplementary Text 4. Selected parent melt composition of Apollo mare basalts

The selected Apollo mare basalts are considered to represent parent melt composition. We have chosen the samples for comparing their formation *P-T* condition with our studied samples on the basis of: (i) Mg# in their bulk composition, (ii) abundance of the REE and their chondrite-normalized patterns, and (iii) from previously chosen parent melts (ref. [23,34,35]). To consider being a parent melt composition, we have chosen the highest bulk Mg# and lowest bulk REE abundance in different groups and subgroups of the Apollo mare basalts. There are high Mg# samples, although those samples are not basaltic rocks and are either clasts within regolith breccias, rake fragments or drill core fragments, which are not considered further as parent melts.

Low- to intermediate-Ti (0-6 wt% TiO_2) mare basalts are found within the collections from Apollo 12 and Apollo 15. In Apollo 12, olivine, pigeonite and ilmenite basalts comprise the three low-Ti varieties. Among the Apollo 12 olivine basalts, 12002 and 12020 are considered non-cumulitic high Mg# (55-57) and have low REE abundances. Previous studies have considered these two samples as representative of parent melt composition[23]. The highest reported bulk Mg# for the pigeonite basalts is lower and the REE abundances are higher when compared to the olivine basalts. Based on petrochemical and textural variations, Apollo 12 pigeonite basalts were suggested to derive from the olivine basalts[36]. In addition, the Apollo 12 pigeonite basalts have also been suggested to have undergone modification either by assimilation or fractional crystallization[34]. The mare basalts of Apollo 15 largely comprise of two varieties: olivine basalts and pigeonite basalts. Among the olivine basalts, 15555 contains high bulk average Mg# (i.e., 48 with the highest value 52) and a low total REE abundance among the olivine basalts. Consequently, we select 15555 as the parent melt composition of Apollo 15 olivine basalts. This sample has previously been suggested as a parent melt composition[23,35]. Schnare et al.[35] proposed that the pigeonite basalts are the fractionated products of the olivine basalts. Among the Apollo 12 ilmenite basalts, sample 12016 shows a non-cumulate texture, highest Mg#, and lowest REE abundances. Therefore, we estimate *P-T* conditions of low-Ti ilmenite basalts from sample 12016. These samples were also selected as parental melt composition by previous studies[23]. Thus, we restrict ourselves to estimate the *P-T* condition of the parent melt of olivine basalts and ilmenite basalts, i.e., 12002, 12020, 12016 and 15555 and compare them with our studied samples (Supplementary Data 4).

Our studies rocks are low-Ti basalts and, as such, we compare them with the low-Ti Apollo basalts. However, we also estimated *P-T* conditions of high-Ti (> 6 wt %TiO_2) mare basalts from the collections of Apollo 11 and Apollo 17. The highest Mg# (51-55) and lowest REE abundances are found in 10050 and 74275 in Apollo 11 and Apollo 17 samples,

respectively. These samples are considered to be non-cumulate parent melts[23]. The estimated *P-T* conditions of these samples are shown in Supplementary Data 4.

Supplementary Text 5. Feasibility of REE modelling and choosing mineral modes of mantle source

Our chemical modeling of REE of the older A-881757 (YAMM), Kalahari 009 and Luna 24 ferrobasalts mantle sources are 75-80 PCS + 1% TIRL (Supplementary Figs. 9 and 14). The REE pattern in these three samples have a distinctness in that they are all LREE depleted (viz., LREE/HREE < 1), and display very small Eu-anomalies (either positive or negative or null). In contrast, mantle sources of the younger NWA 032 and LAP basalts are 86 PCS + 2% TIRL (Supplementary Fig. 9). The younger basalts, which exhibit different REE concentrations and patterns than the older KREEP-free rocks, are somewhat similar to that of the Apollo basalts. As mentioned in the previous section, the REE compositions of the crystalline products (mineral phases) of the LMO are taken from the experimental partition co-efficient values [37-40]. A non-significant difference in REE concentrations and patterns among A-881757 (YAMM), Kalahari 009 and Luna 24 ferrobasalts (Fig. 3) can be attributed to the slight variation in the mantle modal mineralogy in their respective sources (Supplementary Fig. 9). The samples A-881757 (YAMM), Kalahari 009 and Luna 24 ferrobasalts display a very small Eu-anomaly (either positive or negative or null) because of minor quantities of plagioclase in their source(s). It has previously been observed that the size of the Eu-anomaly indicates the plagioclase variation within the source[23,27,32]. Therefore, we note that in a range of 75-80 PCS + 1% TIRL, the source of A-881757 (YAMM), Kalahari 009 and Luna 24 ferrobasalts with slightly variable modal abundances are capable of producing the low variation in Eu-anomaly and the REE pattern among them. In contrast, NWA 032 and LAP basalts show large negative Eu-anomalies, indicating plagioclase undersaturated or very low plagioclase bearing mantle

sources[23,32]. Additionally, the REE patterns in the samples A-881757 (YAMM), Kalahari 009 and Luna 24 ferrobasalt have a distinctness in that they are all LREE depleted (LREE/HREE < 1), unlike some Apollo mare basalts and NWA 032 and LAP basalts (dominantly, LREE/HREE > 1). However, the observed difference in Mg# of A-881757 (YAMM) (Mg# 38-40), Kalahari 009 (Mg# ~49) and Luna 24 ferrobasalts (Mg# ~36) is perhaps suggestive of varying proportions of later crystallized clinopyroxene (pigeonite and augite) and olivine in the mantle source.

Our estimated mantle modal mineralogy ($Ol_{17-30}Opx_{23}Pig_{40-60}Aug_{0-2}Plag_{1-7}$) for the KREEP-free older basalts range is much different for the KREEP-free NWA 032 and LAP basalts with modal mineralogy of $Ol_{10}Opx_{23}Pig_{46}Aug_{20-21}Plag_{0-1}$ (Supplementary Data 3 and Supplementary Fig. 9). Noteworthy to mention here is that our calculated plagioclase abundance in the source of A-881757 (YAMM), Kalahari 009 and Luna 24 ferrobasalts is up to 7%. The plagioclase-bearing mantle source (up to ~8% plagioclase) has been previously suggested for other low-Ti/high-Al basalts such as 12038, 14321, Luna 16 and VLT Luna 24 basalts (refs. [32,41,42]). This shows that the amount of plagioclase varies from source to source and is primarily dependent on the observed Eu-anomaly and the REE pattern. In addition, the measured isotopic abundance in the studied samples also suggest the plagioclase-bearing mantle source (Supplementary Fig. 8). Based on measured high-Al (>11.5 wt.%; Supplementary Data 2) and very low REE abundances in Luna 24 ferrobasalts and Kalahari 009, such compositions would not be consistent with the assimilation of plagioclase rich crustal materials, as Eu abundance and Rb/Sr ratios would show considerable variations. This supports the notion that the aluminous nature of these basalts is a source feature. In the model of LMO crystallization, plagioclase becomes saturated at ~75 PCS, consistent with experimental results of Rapp and Draper[30] and modelling results of Johnson et al.[31]. The variable modal mineralogy

of source and their plagioclase content emphasizes the inherent complexities in the lunar magma ocean and highlights the heterogeneity in the lunar mantle.

The source mineralogy of A-881757 (YAMM), Kalahari 009 and Luna 24 ferrobasalts has more pigeonite compared to early formed olivine and is likely to be more Fe-rich. With the advancement of LMO crystallization, the equilibrium crystallization gave way to fractional crystallization at about 50-70 PCS (refs. [27,28,30,31,43-46]). This would have the effect of forming chemical layering in the upper lunar mantle, the deeper Mg-rich mantle to shallower Fe-rich mantle. Our study shows that the studied samples originated within the shallower mantle than the Apollo basalt melts (e.g., low-Ti 12002, 12020, 15555, and high-Ti 10050, 12016, 74275). However, a limited number of Apollo basalts also exhibit low Mg#, which has been explained through extensive fractionation[23,27,32,34,47]. Therefore, the Fe-rich Apollo basalts might not necessarily represent a shallow source, instead they are fractionated from their parental melt composition. However, their formation *P-T* condition is not retrievable. Therefore, shallow Fe-rich mantle is the most probable mantle source for the low-titanium and REE-depleted YAMM, Kalahari 009 meteorites, and Luna 24 ferrobasalts.

The modal mineralogy and its Fe-rich nature is also supported by experimental and modelling studies following fractional crystallization of TWM composition[30,31]. In fact, our chosen modal source mineralogy also supports some of the remote sensing observation as well. Pyroxene rich mantle at shallow depth (~100 km)[48] has been previously suggested by remote sensing observations within the SPA basin[48,49]. Indeed, the high proportion of pyroxene in the KREEP-free basalt mantle source(s) suggests mantle with pyroxene > olivine (Supplementary Text 5 and 8). Pyroxenite-rich mantle has been previously proposed by Ringwood and Essene[50] based on the analysis of some Apollo 11 basalts.

Supplementary Text 6. Reconciliation of estimated pressure (*P*) and temperature (*T*) for non-KREEP samples

The formation pressure and temperature are estimated following the procedure described in Methods, and the results are provided in Supplementary Data 4 (Fig. 4a and Supplementary Fig. 12). To obtain the liquid composition (Mg#) in equilibrium with the observed Mg# in minerals, we apply the MELTs algorithm to the whole rock composition.

We have chosen the phase diagram mode of pMELTS as a primary method for calculating *P* and *T*. The obtained results are the combined effect of evolving bulk composition and other associated primary minerals rather than any single mineral and/or mineral-melt partitioning. The estimated pressure and temperature obtained from thermobarometry and the pMELTS model show minor differences, depending on the chosen methods. Error in some thermobarometry calculations possibly occurs because the clinopyroxene-liquid and clinopyroxene-only thermobarometry rely on equations that are sensitive to the Al(VI) component and components containing Na. Some of the analysis lacks Na and K data, thus affecting the thermobarometry calculations.

A-881757 and other YAMM meteorites

We selected pyroxene cores with Mg# (50-54). A phase diagram mode of pMELTS obtains the *P* and *T* for the composition similar to the pyroxene cores between 0.3-0.8 GPa and 1100-1190°C. This range lies between estimates from clinopyroxene-liquid thermobarometry, 0.5-0.6 GPa and 1140-1160°C, and clinopyroxene-only thermobarometry, 0.4-0.8 GPa, and 1100-1120°C, assuming the standard error of estimate (SEE) in each method. Accounting results from all the methods, the *P* and *T* of the YAMM meteorite clan range within 0.3-0.8 GPa and 1100-1190°C, respectively (Supplementary Data 4).

Kalahari 009

The data for whole-rock and mineral composition of Kalahari 009 were taken from Sokol et al.[11]. Using the highest Mg# olivine and pyroxene composition, a pMELTS phase diagram mode calculates *P* and *T* within the range of 0.7-0.8 GPa and 1215-1235°C. Using an appropriate liquid composition, which is in equilibrium with the observed minerals, the olivine-liquid thermometry results show a temperature range of 1195-1225°C, while clinopyroxene-liquid yields *P-T*, 0.7-1.0 GPa and 1215-1235°C and clinopyroxene-only estimate P-T between 0.8-1.0 GPa and 1195-1225°C. The broad range of *P-T* for Kalahari 009 lies within 0.7-1 GPa and 1195-1235°C (Supplementary Data 4).

Luna 24 ferrobasalt

The Luna ferrobasalt composition taken from Vaniman and Papike[13] is used for thermobarometric calculations. The clinopyroxene-only thermobarometer yields an estimated formation pressure of ~ 0.6 GPa and temperature of ~ 1155°C. This result is close to the experimental value, 1180°C and 0.4-0.5 GPa[20].

NWA 032

The *P-T* for NWA 032 was estimated from the whole rock and mineral data of Fagan et al.[15]. The phase diagram mode for the NWA bulk composition calculates a formation temperature of 1170-1190°C at a pressure of 0.3-0.5 GPa. The olivine-liquid thermometry on the pressure range obtained using the phase diagram method shows a formation temperature of 1180-1200°C. Due to the lack of clinopyroxene data, we could not apply other thermobarometry methods.

LAP basalts

Compositions of LAP basalts data were taken from Day et al.[17]. The results of the phase diagram method on the bulk of LAP 02205 suggest formation *P-T* of 0.5-0.6 GPa and 1180-1200°C, respectively. We perform thermobarometry on the minerals with the most magnesian olivine and pyroxenes. The results of olivine thermometry for pMELTS estimated pressure suggests a formation temperature range of 1170-1180°C. The estimated temperature is 1160°C at 0.43 GPa, based on clinopyroxene-only thermobarometry for pyroxenes of LAP 03632 basalt (Supplementary Data 4). The obtained results are similar to the experiments (1150-1200°C at <0.6 GPa) performed by Elardo et al.[51].

Supplementary Text 7. Calculated *P-T* of the Apollo mare basalts

To be internally consistent, we have performed the *P-T* calculation by pMELTS and thermobarometry of the parental melt compositions (e.g., low-Ti 12002, 12020, 15555, and high-Ti 10050, 12016, 74275; Supplementary Text 4) of different Apollo sample suites. The obtained results for these samples suggest that the observed differences in the *P-T* conditions of our studied rocks and the Apollo mare basalts are real as there is no method bias associated with our models (Supplementary Data 4). This study further demonstrates that the formation *P-T* obtained by pMELTS closely matches the results from multiple saturation point (MSP) experiments of Apollo samples. For example, we calculate ~ 1.0 GPa and ~ 1350°C for 15555, while earlier experiments by Kesson et al.[52] and Walker et al.[53] deduced 1.0-1.2 GPa and 1300-1350°C for the same sample. Several recent studies compare the model (such as pMELTS, MAGPOX, and Perple_X) calculations with the MSP experiments in detail[54,55]. These studies have found their results are close to experimental values. In fact, the time-consuming and expensive experiments show a single datum in *P-T-X* space, while the equilibrium

thermodynamic models offer a mean to extrapolate the experimental results seamlessly across various composition and *P*-*T* space.

Supplementary Text 8. Geochemical comparison between low-Mg Apollo basalts and KREEP-free basalts

In common with studied KREEP-free basalts, some Apollo mare basalts also show low-Mg values. However, this difference could simply arise from the fact that low-Mg Apollo basalts are fractional crystallized product of their high-Mg counter-part which are thought to come from an olivine-rich mantle source as opposed to pyroxene-rich mantle sources for KREEP-free basalts (Supplementary Text 4 and 5). We therefore explore the difference in Apollo mare basalts and KREEP-free basalts that could arise from differences in their mantle source lithology.

Melts from the pyroxenite sources of the Earth are suggested to have higher Ni content at a given bulk MgO content when compared to their peridotite-sourced counterparts[56-58] due to the retention of Ni in olivine. Several recent studies such as Yang and Zhou[59] and Yang et al.[60] utilize combination of ratios such as FC3MS value ($FeO/CaO-3*MgO/SiO_2$, all in wt.%) and FCKANTMS (FCKANTMS = $\ln(FeO/CaO) - 0.08 * \ln(K_2O/Al_2O_3) - 0.052 * \ln(TiO_2/Na_2O) - 0.036 * \ln(Na_2O/K_2O) * \ln(Na_2O/TiO_2) - 0.062 * (\ln(MgO/SiO_2))^3 - 0.641 * (\ln(MgO/ SiO_2))^2 - 1.871 * \ln(MgO/SiO_2) - 1.473$, all the major elements in wt.%) to discriminate between peridotite and pyroxenite sources.

We plot bulk Ni (in ppm), Al_2O_3 (in wt. %), FC3MS and FCKANTMS against bulk MgO (in wt. %) (Supplementary Fig. 11). In Supplementary Fig. 11c and 11d, we see that the pyroxene-rich mantle-sourced KREEP-free basalts show a small cluster with value higher than most olivine-rich mantle-sourced low-Mg Apollo mare basalts similar to terrestrial pyroxenites[59,60]. Some low-Mg Apollo samples (mainly Apollo 11 and few Apollo 12 mare

basalts) plot in the same field to KREEP-free basalts while some samples such as Kalahari 009, plot away from the cluster possibly because of differences in their source compositions and degree of melting.

Nonetheless, we do not consider these variations to result from pyroxenite sources like those envisaged for Earth. This is because initial Ni contents and fO_2 conditions of the lunar mantle are highly distinct from those of the terrestrial mantle[61].

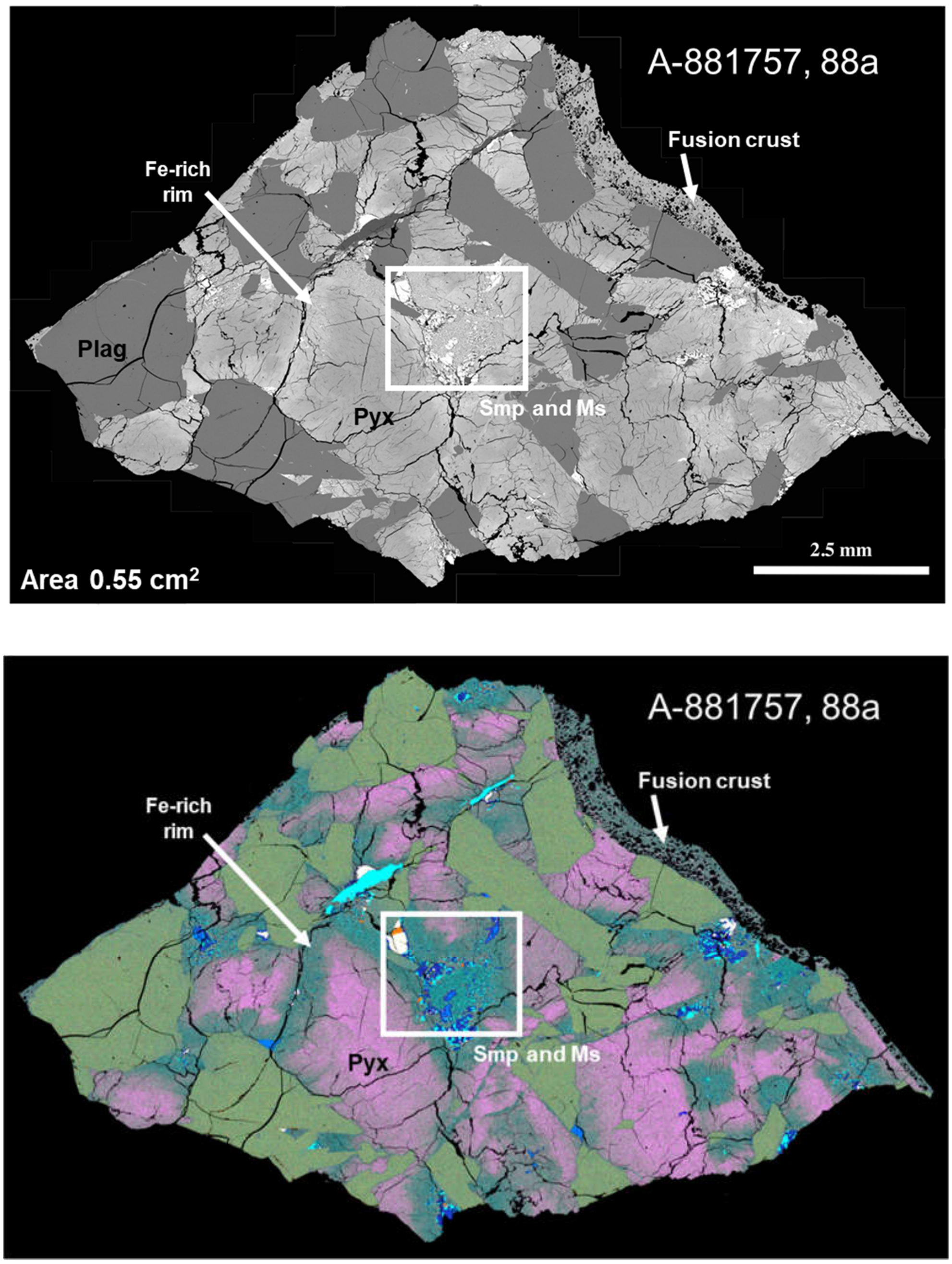

Supplementary Fig. 1│ BSE (left panel) and X-ray composite (right panel) images of studied section A-881757 (area ~0.55 cm^2). Coarse pyroxene and plagioclase along with symplectite and mesostasis (white box) in A-881757. Combined X-ray image highlights the compositional variation within pyroxenes and late-phase mineral assemblage. Abbreviations used are Pyx: Pyroxene; Plag: Plagioclase; Smp: Symplectites; Ms: Mesostasis. Composite image is made using freely available Image J software (https://imagej.nih.gov/ij/download.html). The merged colors are selected as Green-Al, Blue-Fe, Magenta- Mg, Cyan-Si, Yellow-P, Red-Ca, and Gray-Ti.

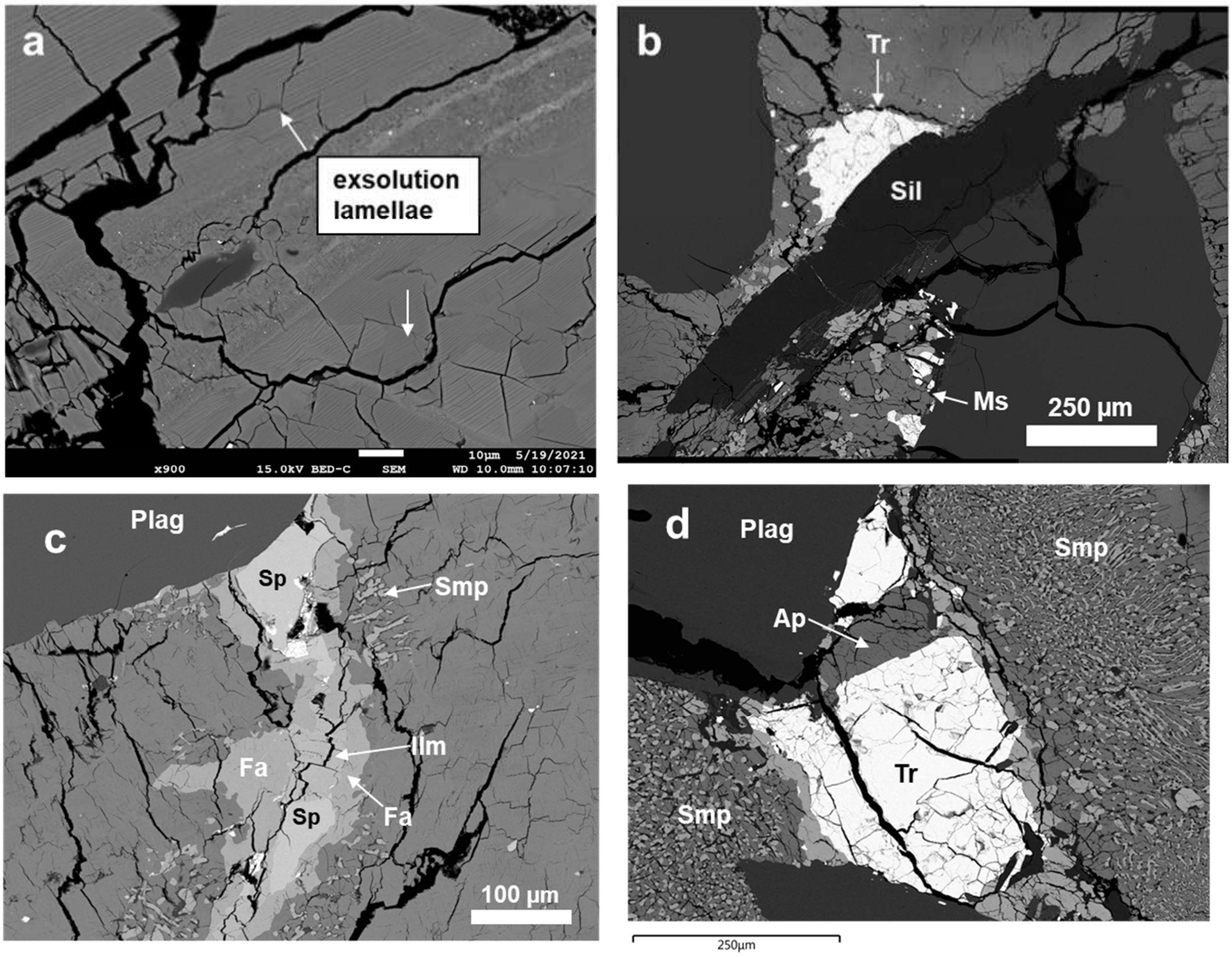

Supplementary Fig. 2 | Exsolved pyroxenes and the textural relationship among late phase assemblages in A-881757. **a** Fine exsolution lamellae (~1 µm) in pyroxene at 10 µm resolution. **b** Occurrence of elongated silica along with troilite in the mesostasis. **c** spinels occurring along with ilmenite further rimmed by fayalitic olivine. **d** troilite and apatite occurring within the symplectites. Abbreviations used are Tr: troilite; Sil: silica; Ms: mesostasis; Sp: spinel; Fa: fayalite; Ilm: ilmenite; Smp: symplectite; Plag: plagioclase and Ap: apatite.

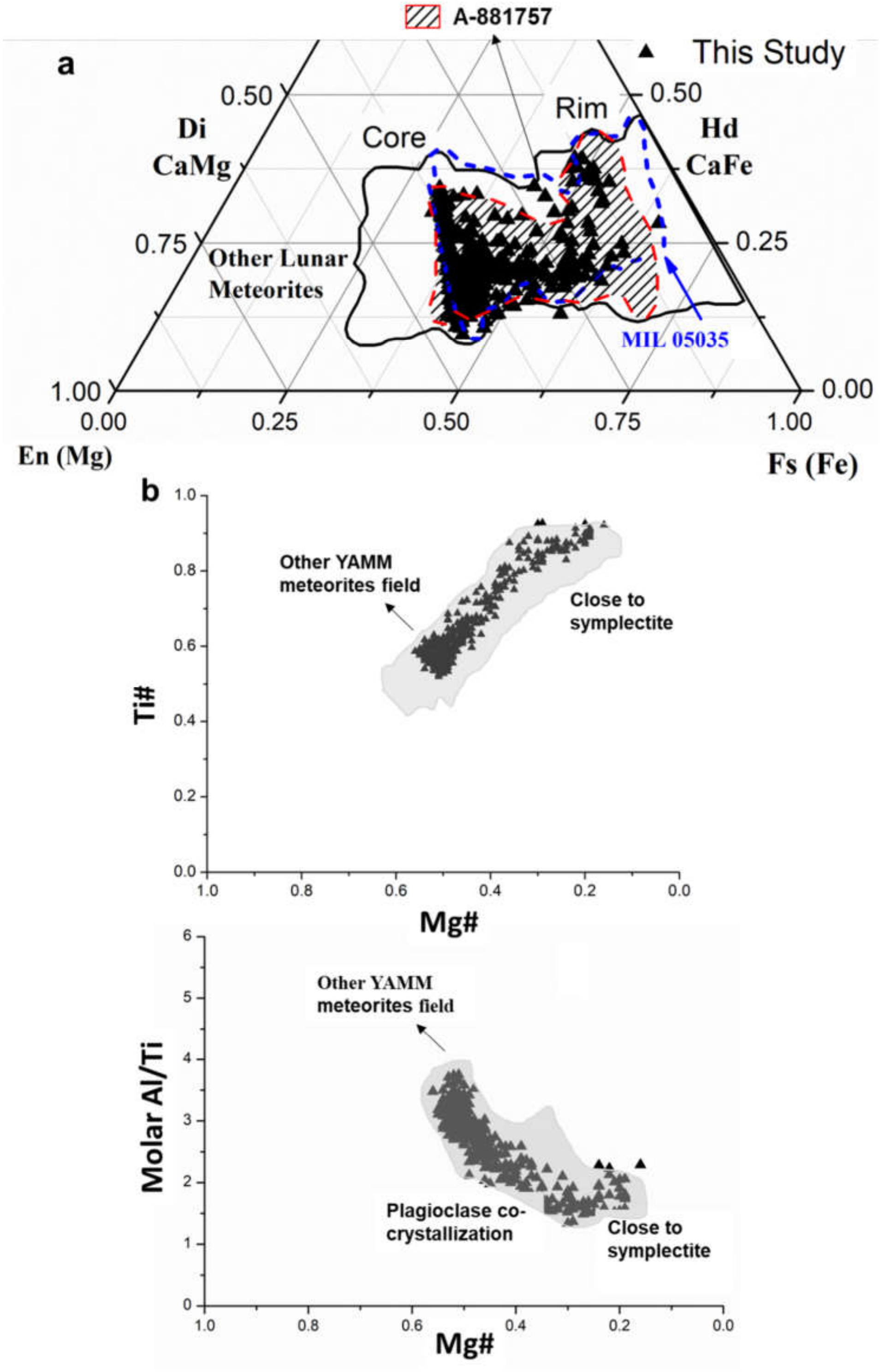

Supplementary Fig. 3│ Pyroxene mineral composition for A-881757. In **a** En-Fs-Wo components of pyroxenes are plotted. The shaded field is previously measured A-881757 pyroxene compositions (ref.[62,63]). The blue dashed lines correspond to MIL 05035 pyroxenes (refs. [4,5]). The black outlined field corresponds to the composition measured for other lunar meteorites. E.g., Swayyah 001 (ref. [64]), Ferroan Gabbro in NWA – 773, 2727, 7007, 3160, 3170 (refs. [64.65]), MET 01210 (ref. [1]). **b** The pyroxene molar Ti# (Ti/Ti+Cr) vs. Mg# (Mg/Mg+Fe) and molar Al/Ti vs. Mg# are plotted (Supplementary Text 1).

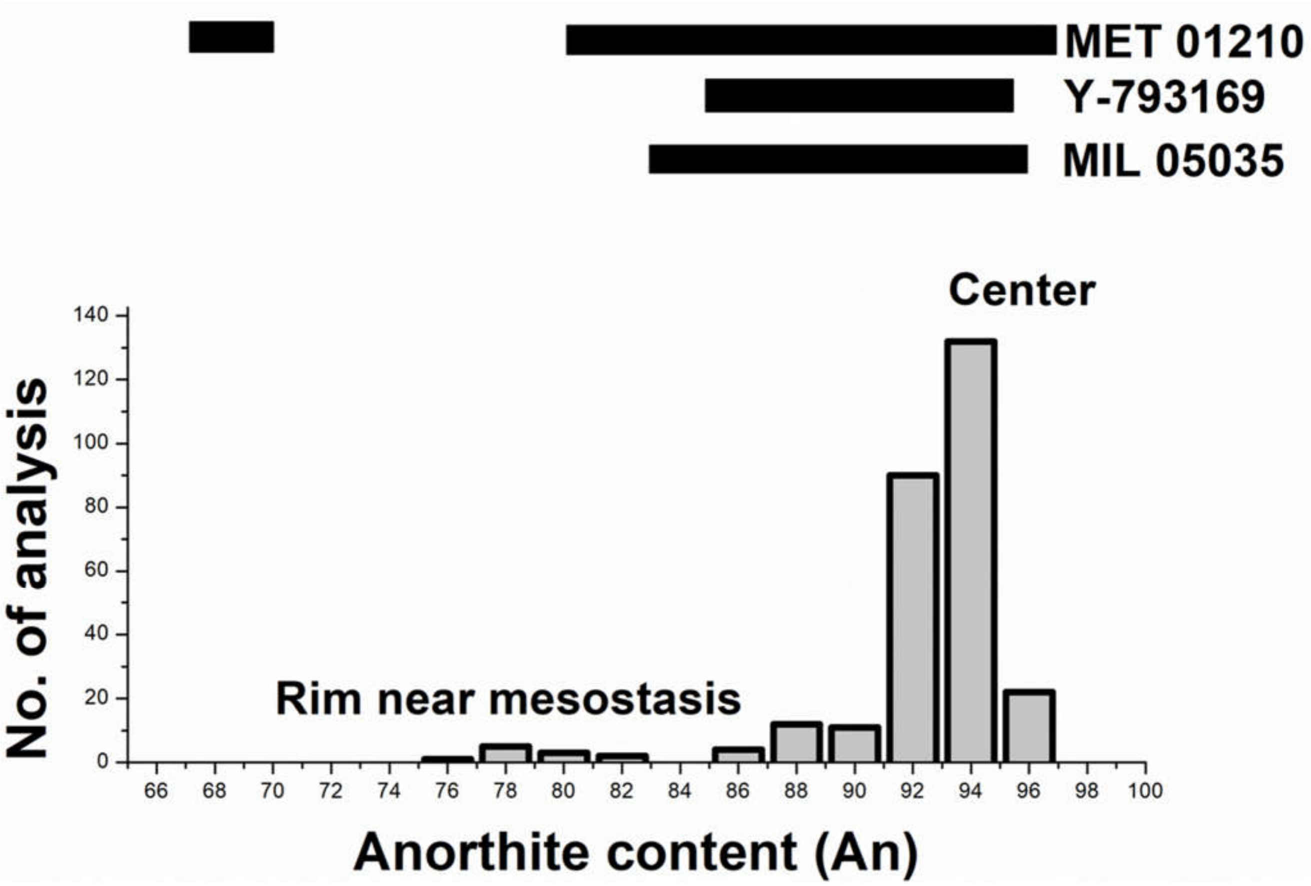

Supplementary Fig. 4│ Anorthite (An) content variation in plagioclase of A-881757 compared to other clan meteorites. Data sources are MET 01210: refs. [1,66]; Y-793169: ref. [6]; MIL 05035: refs. [4,5]; A-881757: this study.

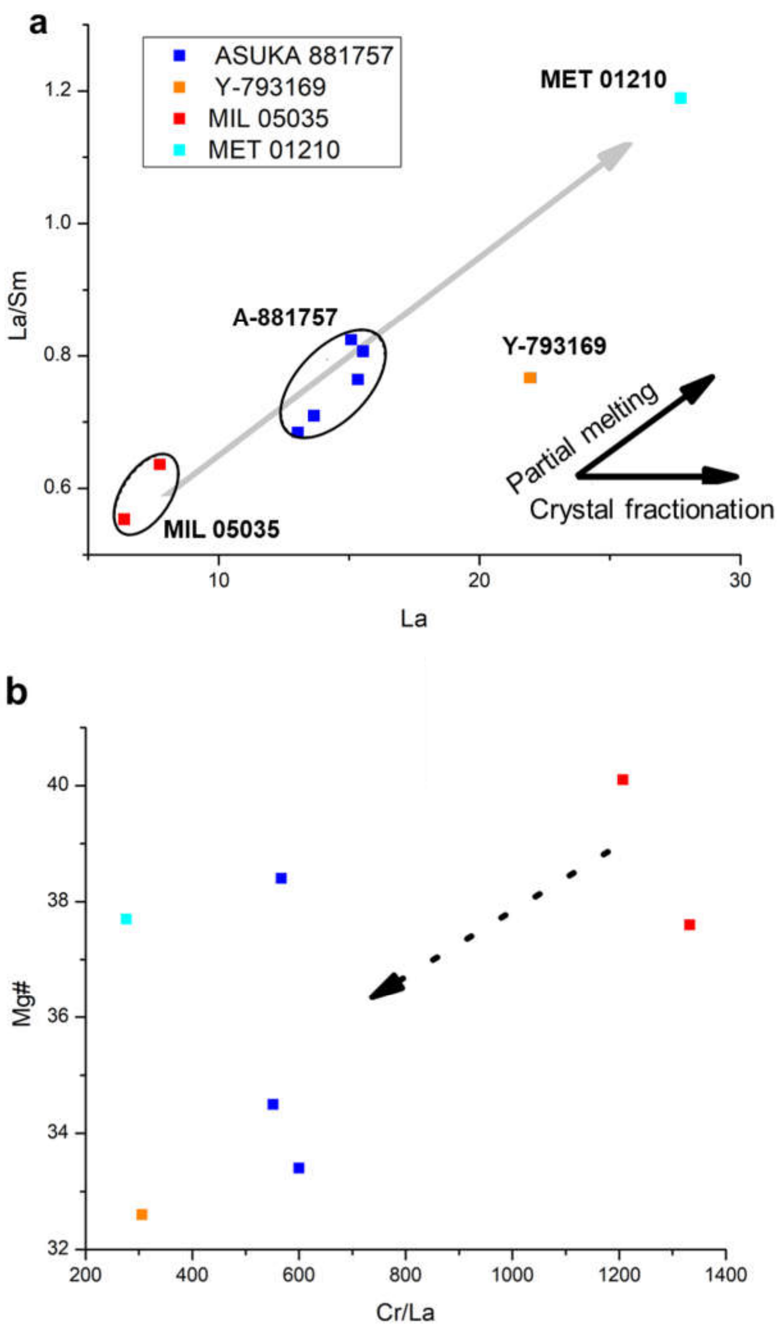

Supplementary Fig. 5│ Plot showing partial melting relation of YAMM meteorites and primitive nature of MIL 05035. a La/Sm versus La plot for YAMM meteorites, MIL 05035, A-881757, MET 01210 show a partial melting relation while Y-793169 shows signs of fractional crystallization. **b** Mg# plotted against Cr/La measured in the YAMM meteorites. The high Cr/La ratio in MIL 05035 suggests it to be the most primitive among YAMM clan members. Data sources are provided in Supplementary Data 2.

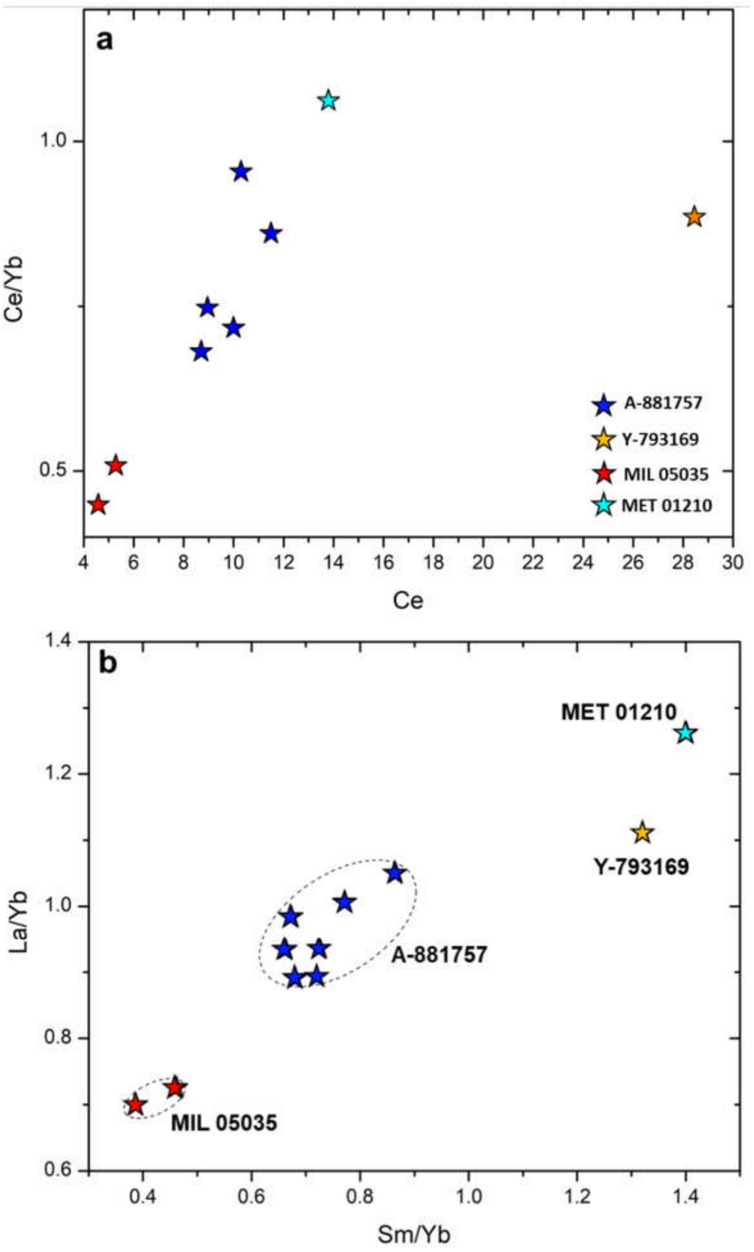

Supplementary Fig. 6 | Plot showing partial melting relation of YAMM meteorites using trace element ratios. a Ce/Yb versus Yb plot for YAMM meteorites, MIL 05035, A-881757, MET 01210 show a partial melting relation while Y-793169 shows signs of fractional crystallization. **b** La/Yb vs Sm/Yb measured in the YAMM meteorites showing a partial melting relation in the YAMM clan. Data sources are the same as given in Supplementary Data 2.

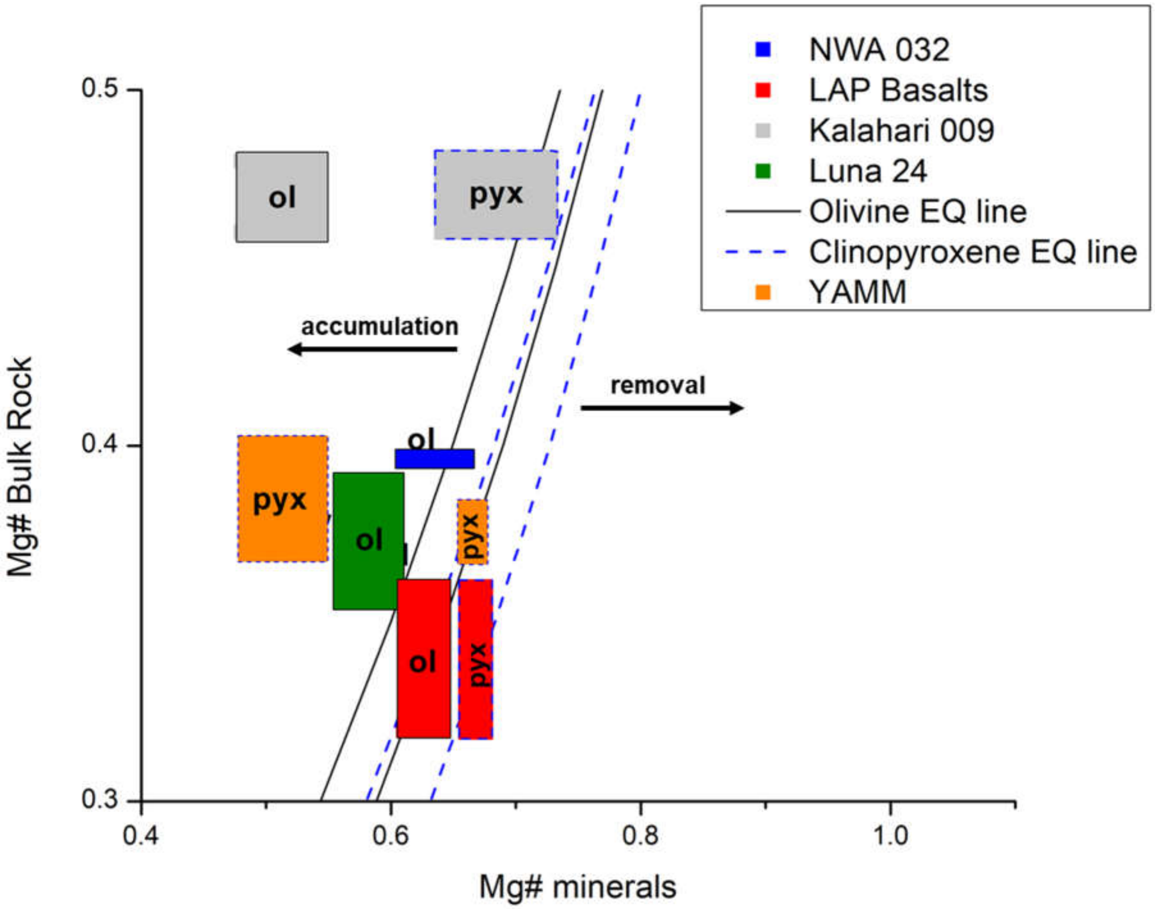

Supplementary Fig. 7 | Plot of Mg# of the bulk rock versus Mg# of the olivine and pyroxene in different KREEP-free rocks (similar to Rhodes diagram of ref. [71]). Black and dashed-blue lines represent olivine-basaltic melt $Kd_{Fe\text{–}Mg}$ = 0.32 ± 0.03 (ref. [25]) and clinopyroxene-basaltic melt $Kd_{Fe\text{–}Mg}$ = 0.28 ± 0.03 (ref. [26]), respectively, indicating equilibrium olivine and pyroxene composition with basalt. The bulk compositions of Luna 24 ferrobasalts, NWA 032, and LAP basalts are in equilibrium with the observed olivine and clinopyroxene composition, suggesting the samples approach a melt composition. However, Kalahari 009 and YAMM basalts show some signs of olivine and pyroxene accumulation (Supplementary Text 3). The data sources are the same as in Fig. 1.

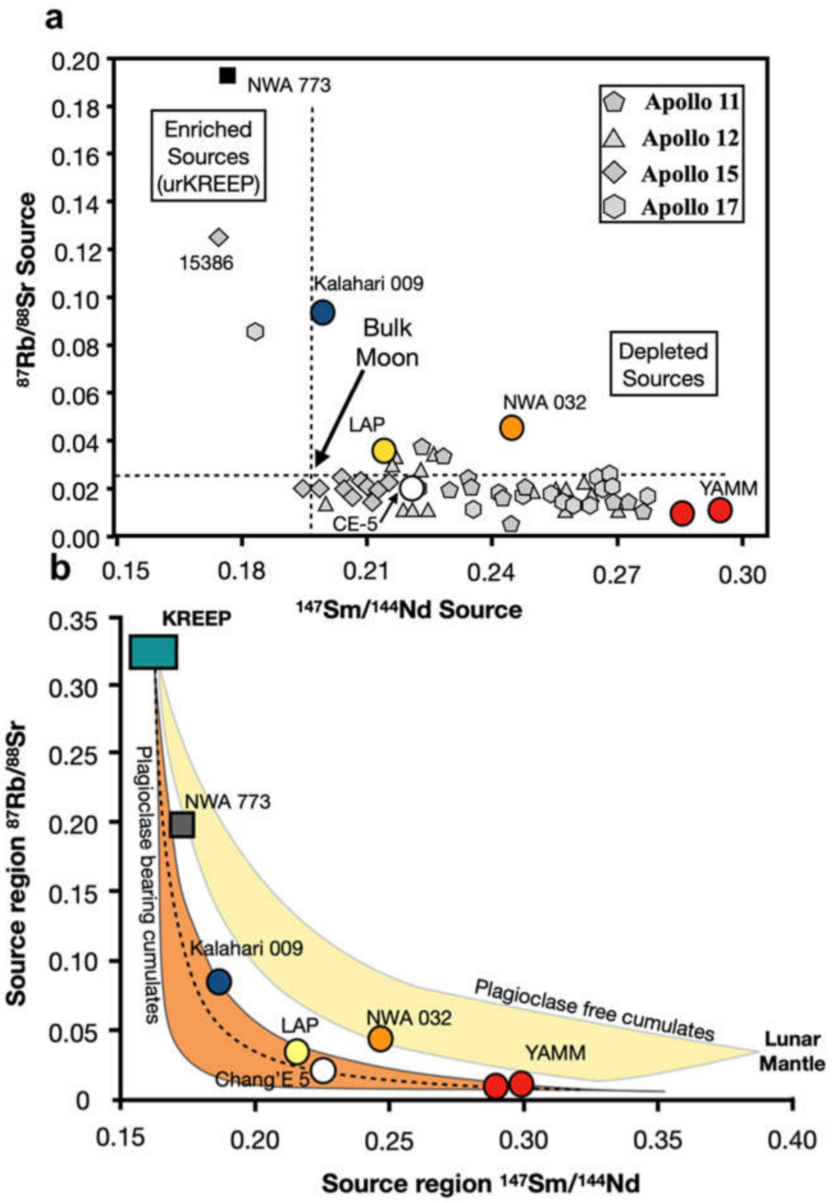

Supplementary Fig. 8 | Calculated $^{87}Rb/^{86}Sr$ versus $^{147}Sm/^{144}Nd$ for source regions of Apollo mare basalts and meteorites. a Illustration of the relationship of KREEP-free to KREEP-related samples (after ref. [18]). YAMM basalts (MIL 05035 and A-881757) are the most depleted variety among the lunar samples. **b** The figure describes the mixing line of the KREEP (adopted from refs. [18]). Most non-KREEP samples plot far away from the KREEP basalts providing evidence of an unassimilated source. Data Source: Plot of $^{87}Rb/^{86}Sr$ versus $^{147}Sm/^{144}Nd$ of lunar basalt source regions calculated using a single-stage model with an initial lunar $^{87}Sr/^{86}Sr$ ratio of LUNI = 0.69903 (refs. [67,68]) and considering the age of the Moon as 4558 Ma. The $^{147}Sm/^{144}Nd$ ratios of lunar basalt sources are calculated assuming a two-stage chondritic evolution. In this model, the Moon follows a path of chondritic evolution until 4.42 Ga and then differentiates, forming the source regions of lunar basalts (refs. [69,70]). Data are from the same sources as given in Fig. 2.

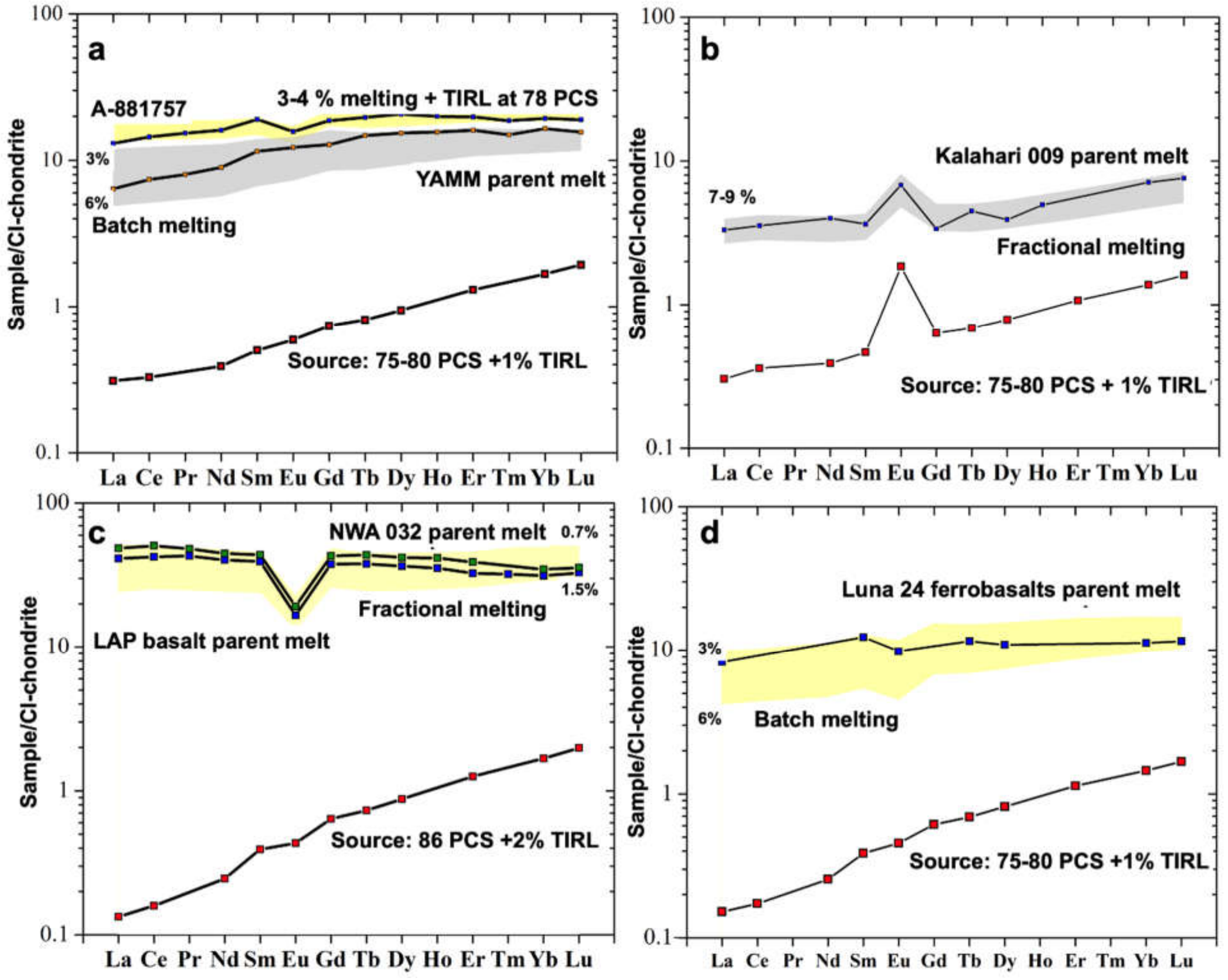

Supplementary Fig. 9│ Source composition deduced from trace element modeling for YAMM and other KREEP-free basalts. **a** Grey area denotes the melt produced by 4-6% partial melting of the mantle source (78 PCS + 2 TIRL; PCS: percent crystallized solid; TIRL: trapped instantaneous residual liquid). Mineral modes in the source are assumed to be 40 vol. % olivine, 40 vol. % orthopyroxene, 19 vol. % pigeonite, and ~1 vol. % plagioclase. A-881757 similarly can be produced by 3-4% partial melting with presence of trapped liquid at 78 PCS. **b** Grey area denotes the melt produced by 7-9% partial melting of mantle source at 78 PCS + 1% TIRL with assumed modal mineralogy of 52 vol. % olivine, 23 vol.% orthopyroxene, 17 vol. % pigeonite, and ~8 vol. % plagioclase. **c** Yellow area highlights the melt produced by very low (0.7–1.5%) partial melting of mantle source (86 PCS + 2% TIRL) with assumed mineral mode of 52 vol. % olivine, 23 vol. % orthopyroxene, 23 vol. % pigeonite, and 2 vol.% augite for NWA 032 and 52 vol. % olivine, 23 vol. % orthopyroxene, 23 vol. % pigeonite, ~1 vol. % augite and ~1 vol. % plagioclase for LAP basalts. **d** yellow area indicates the melt produced by 3-6% partial melting of mantle source (78 PCS + 1% TIRL) with assumed mineralogy of 48 vol. % olivine, 23 vol. % orthopyroxene, 23 vol. % pigeonite, 3 vol. % augite and 3 vol. % plagioclase. The additional details are described in Methods, Supplementary Text 3 and Supplementary Data 3.

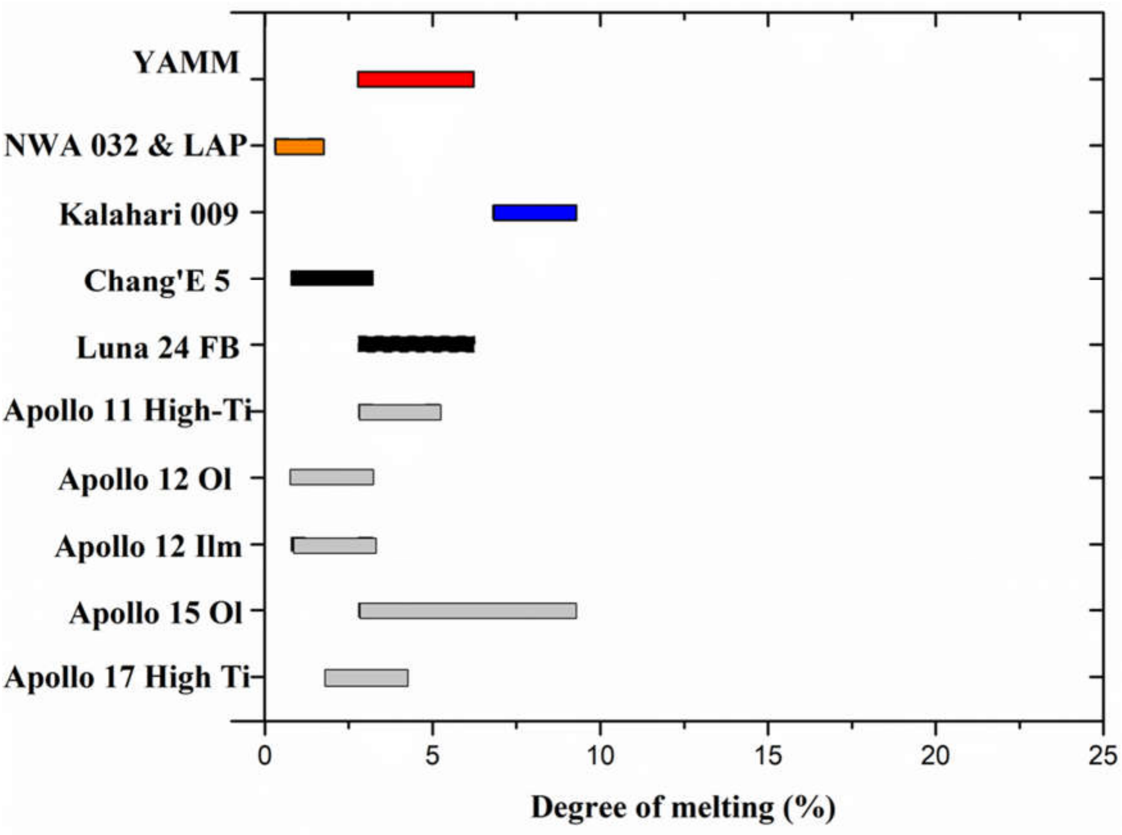

Supplementary Fig. 10 | The calculated degree of melting for YAMM and other KREEP-free rocks compared to Apollo mare basalts using trace element modeling. Data for degree of melting of the Apollo mare basalts are from ref. [23], and the remaining samples are from this study (Methods and Supplementary Text 3).

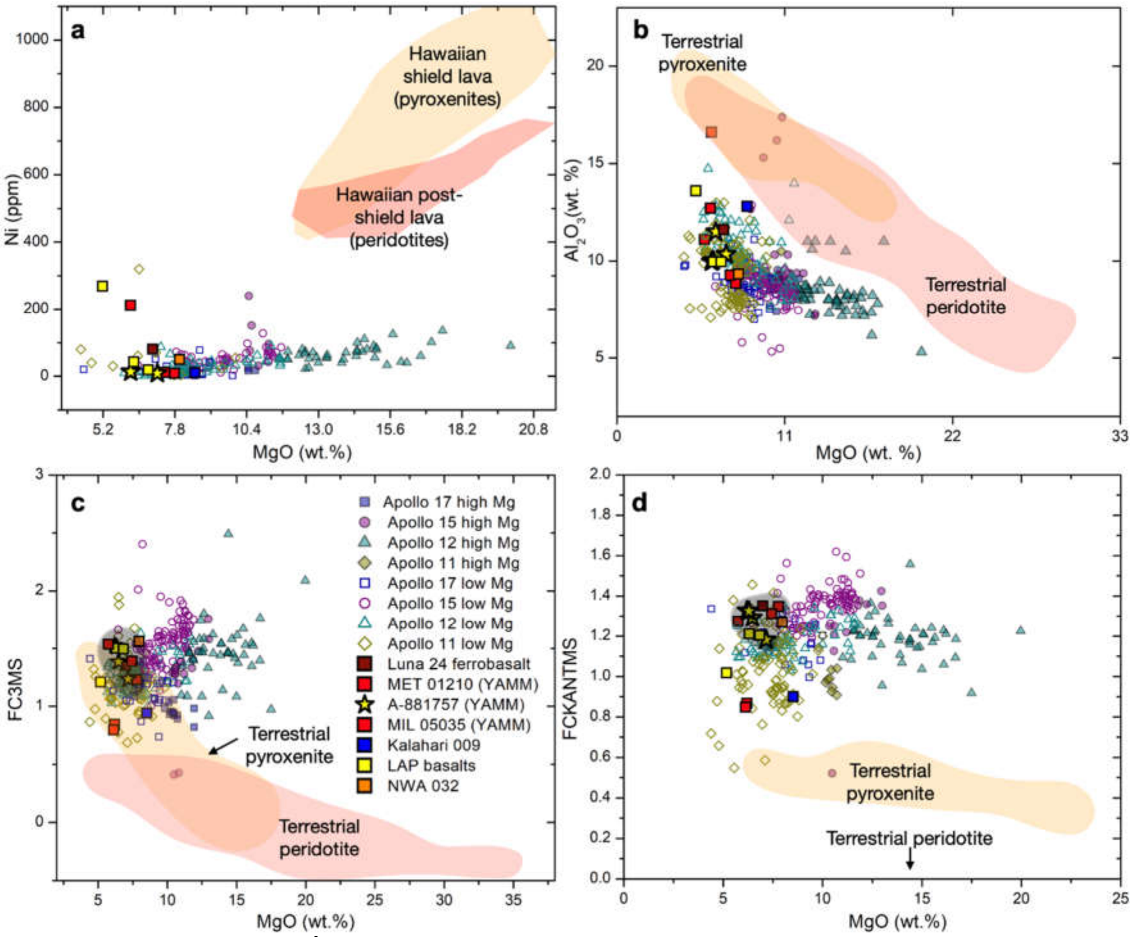

Supplementary Fig. 11 | The range of KREEP-free basalts plotted against various terrestrial pyroxenite source markers. FC3MS ($FeO/CaO-3*MgO/SiO_2$, all in wt.%) and FCKANTMS ($\ln(FeO/CaO) -0.08*\ln(K_2O/Al_2O_3) -0.052*\ln(TiO_2/Na_2O) - 0.036*\ln(Na_2O/K_2O) * \ln(Na_2O/TiO_2) - 0.062*(\ln(MgO/SiO_2))^3 - 0.641*(\ln(MgO/SiO_2))^2 - 1.871*\ln(MgO/SiO_2) - 1.473$) show most prominent distinction (grey cluster) between KREEP-free basalts and low-Mg Apollo samples. The cutoff between low-Mg and high-Mg is kept as Mg# 49 (Supplementary Text 8). **a** plot showing variation of bulk Ni (ppm) with MgO (wt. %) for high- and low-Mg Apollo samples along with KREEP-free basalts. The regions highlighted in yellow and red show terrestrial pyroxenite and peridotite composition of Hawaiian lavas taken from Sobolev et al.[56]. **b** The Al_2O_3 versus MgO relation showing non-distinctive trend in KREEP-free basalts and low-Mg Apollo basalts. The yellow and red highlighted regions are terrestrial pyroxenite and peridotite melt compositions from Yang et al.[72], respectively. **c** FC3MS versus MgO plot showing small cluster (grey shaded) of KREEP-free basalts having high FC3MS value for a particular MgO value than most low-Mg Apollo basalts, similar to terrestrial settings. The yellow and red highlighted regions are terrestrial pyroxenite and peridotite melt compositions from Yang et al.[72], respectively. **d** FCKANTMS log ratio plotted against MgO show small clustering for KREEP-free basalts with high FCKANTMS value for a given MgO value when compared to most low-Mg Apollo mare basalts. The yellow highlighted region shows terrestrial pyroxenite composition from Yang et al.[60]. Data Sources: Supplementary Data 2 and Mare Basalt Database[78].

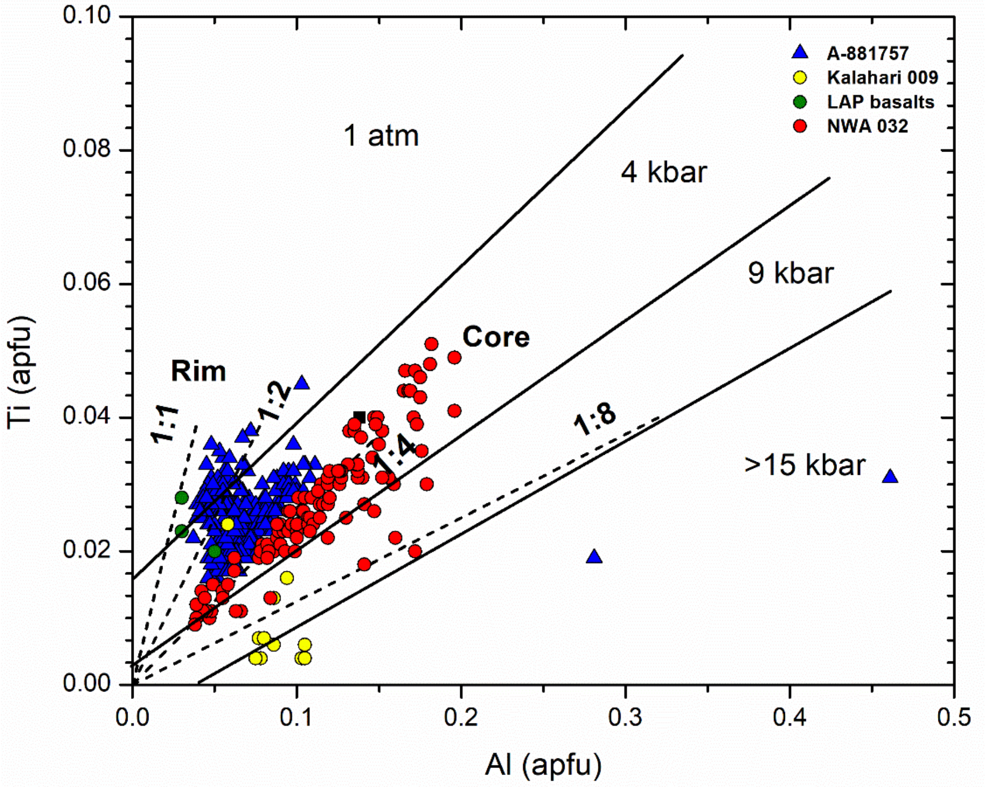

Supplementary Fig. 12 | Al-Ti geobarometer in clinopyroxene of KREEP-free basalts. The plots of molar Al versus Ti of pyroxenes in studied rocks over the pressure-sensitive partitioning lines of Al and Ti in clinopyroxene. The calibrated partition lines are experimentally-derived from controlled high-pressure (ref. [73]). The estimated pressure shows results similar to the phase diagram mode of pMELTS and thermobarometry (Methods and Supplementary Text 4).

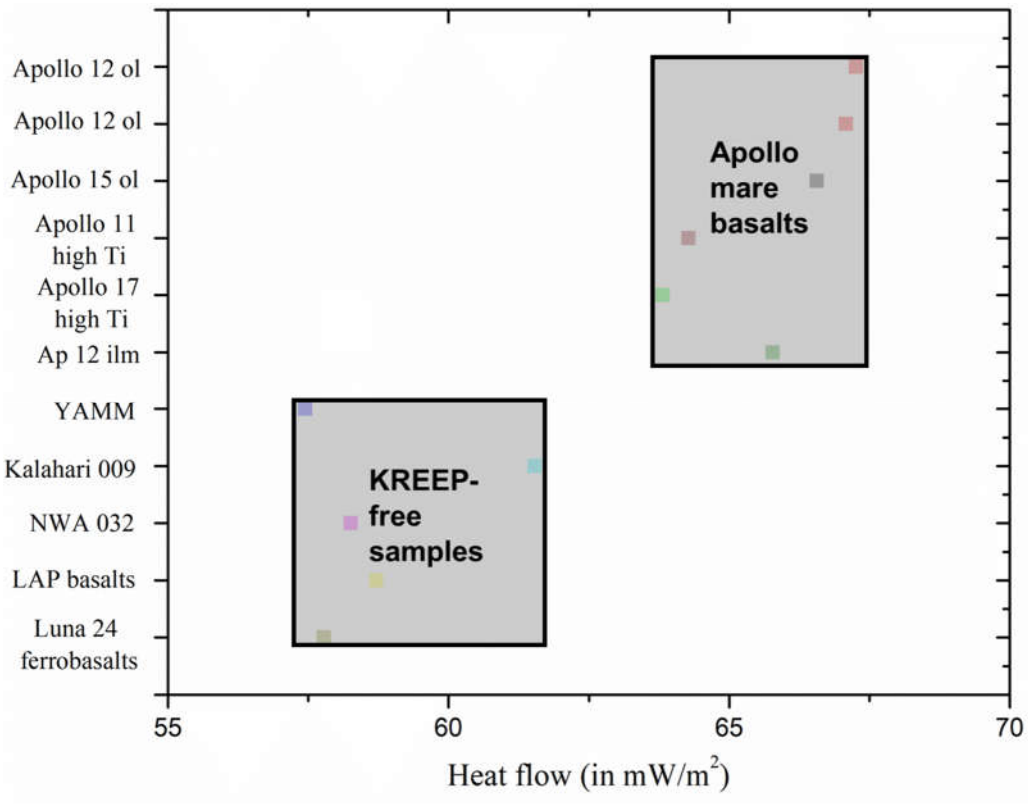

Supplementary Fig. 13 | Surface heat flow obtained assuming the crust-mantle boundary at 40 km depth. The calculation was performed using compositional data of YAMM, Kalahari 009, NWA 032, LAP basalts, and Luna 24 ferrobasalts. The *P-T* and degree of melting estimates for KREEP-free basalts are described in Methods, Supplementary Text 7 and Supplementary Data 4. Data for Apollo mare basalts degree of melting from ref. [23].

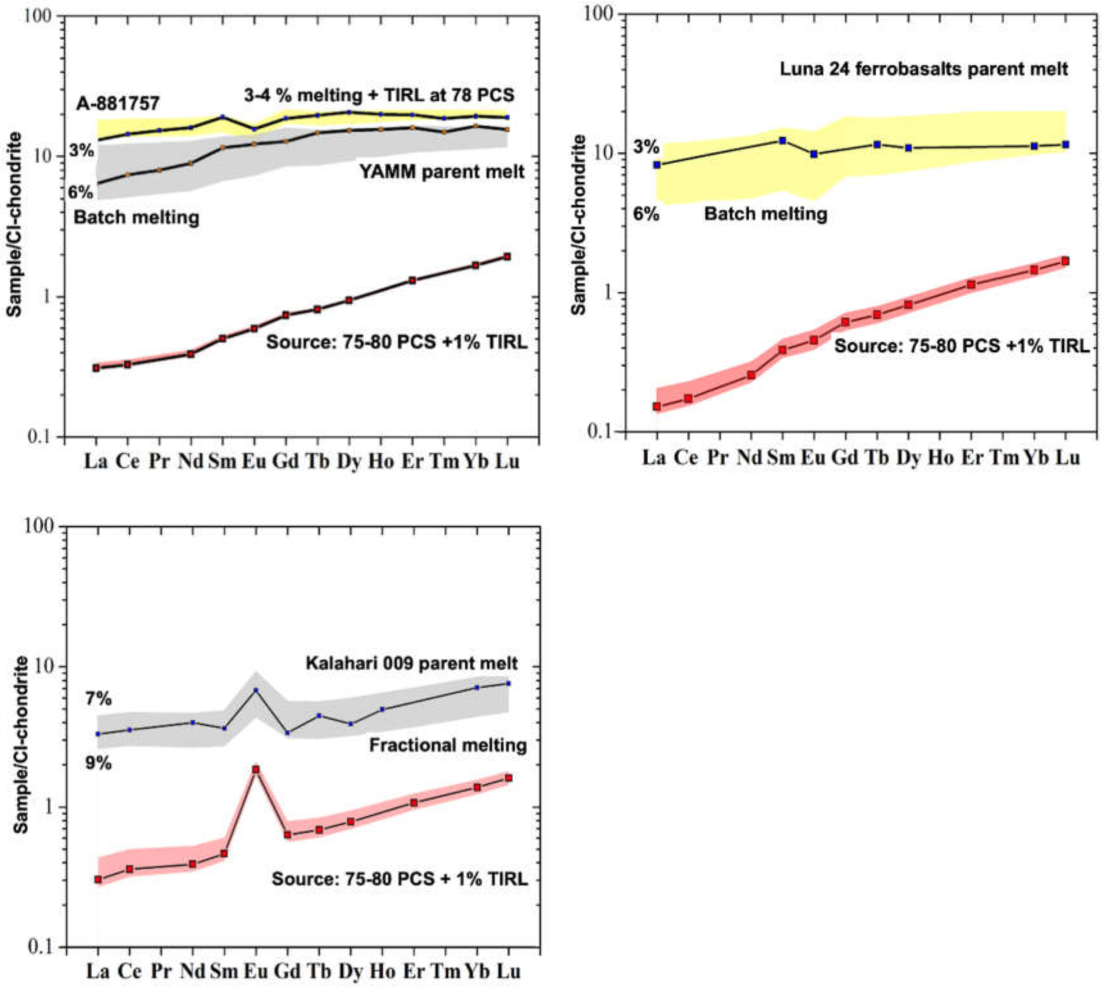

Supplementary Fig. 14 | Plot showing minimal variation observed in changing 75-80 PCS for a fixed modal mineralogy. The area highlighted in red is showing the range (75-80 PCS) of mantle source composition that can produce the observed REE pattern in the YAMM basalts, Kalahari 009 and Luna 24 ferrobasalts (Supplementary Text 3 and 5).

Supplementary Table 1 | The mineral REE partition coefficients used in model calculations.

	Olivine[37]	Orthopyroxene[38]	Augite[38]	Pigeonite[39]	Plagioclase[40]
La	0.0001	0.0007	0.0446	0.0009	0.0418
Ce	0.0001	0.0015	0.0733	0.0017	0.0302
Pr					
Nd	0.0001	0.0055	0.1544	0.0058	0.0236
Sm	0.0006	0.0143	0.251	0.011	0.017
Eu	0.0007	0.0204	0.2952	0.0068	1.2
Gd	0.001	0.0281	0.3377	0.021	0.0105
Tb	0.002	0.0376	0.3758	0.027	0.0095
Dy	0.003	0.0487	0.4071	0.034	0.0089
Ho					
Er	0.008	0.0714	0.4402	0.055	0.0077
Tm					
Yb	0.019	0.0913	0.4426	0.087	0.0065
Lu	0.03	0.0995	0.4368	0.11	0.0068

Supplementary References

1. Day, J. M. D., Floss, C., Taylor, L. A., Anand, M. & Patchen, A. D. Evolved mare basalt magmatism, high Mg/Fe feldspathic crust, chondritic impactors, and the petrogenesis of Antarctic lunar breccia meteorites Meteorite Hills 01210 and Pecora Escarpment 02007. *Geochim. Cosmochim. Acta* **70**, 5957–5989 (2006).
2. Arai, T. et al. Antarctic lunar meteorites Yamato-793169, Asuka-881757, MIL 05035, and MET 01210 (YAMM): Launch pairing and possible cryptomare origin. *Geochim. Cosmochim. Acta* **74**, 2231–2248 (2010).
3. Yanai, K. Gabbroic meteorite Asuka-31-Preliminary examination of a new type of lunar meteorite in the Japanese collection of Antarctic meteorites. *Proc. Lunar Planet. Sci.* **21**, 317–324 (1991).
4. Liu, Y., Floss, C., Day, J. M. D., Hill, E. & Taylor, L. A. Petrogenesis of lunar mare basalt meteorite Miller Range 05035. *Meteorit. Planet. Sci.* **44**, 261–284 (2009).
5. Joy, K. H. et al. The petrology and geochemistry of Miller Range 05035: A new lunar gabbroic meteorite. *Geochim. Cosmochim. Acta* **72**, 3822–3844 (2008).
6. Takeda, H., Arai, T. & Saiki, K. Mineralogical studies of lunar meteorite Yamato-793169, a mare basalt. *Antarct. Meteor. Res.* **6**, 3 (1993).
7. Mikouchi, T. Shocked plagioclase in the lunar meteorites Yamato-793169 and Asuka-881757: Implications for their shock and thermal histories. *Antarct. Meteor. Res.* **12**, 151 (1999).
8. Misawa, K., Tatsumoto, M., Dalrymple, G. B. & Yanai, K. An extremely low UPb source in the Moon: U- Th-Pb, Sm-Nd, Rb-Sr, and 40Ar-39Ar isotopic systematics and age of lunar meteorite Asuka 881757. *Geochim. Cosmochim. Acta* **57**, 4687–4702 (1993).
9. Torigoye-Kita, N., Misawa, K., Dalrymple, G. B. & Tatsumoto, M. Further evidence for a low U/Pb source in the moon: U-Th-Pb, Sm-Nd, and Ar-Ar isotopic systematics of lunar meteorite Yamato-793169. *Geochim. Cosmochim. Acta* **59**, 2621–2632 (1995).
10. Nyquist, L. E., Shih, C. Y. & Reese, Y. D. Sm-Nd and Rb-Sr Ages for MIL 05035: Implications for Surface and Mantle Sources. in *38th Annual Lunar and Planetary Science Conference* 1702 (2007).
11. Sokol, A. K. et al. Geochemistry, petrology and ages of the lunar meteorites Kalahari 008 and 009: New constraints on early lunar evolution. *Geochim. Cosmochim. Acta* **72**, 4845–4873 (2008).
12. Terada, K., Anand, M., Sokol, A. K., Bischoff, A. & Sano, Y. Cryptomare magmatism

4.35 Gyr ago recorded in lunar meteorite Kalahari 009. *Nature* **450**, 849–852 (2007).

13. Vaniman, D. T. & Papike, J. J. Ferrobasalts from Mare Crisium: Luna 24. *Geophys. Res. Lett.* **4**, 497–500 (1977).
14. Ma, M. S. et al. Chemistry and petrology of Luna 24 lithic fragments and <250 μm soils: constraints on the origin of VLT mare basalts. in *Mare Crisium: The view from Luna 24* (eds. Merrill, R. B. & Papike, J. J.) 569–592 (1978).
15. Fagan, T. J. et al. Northwest Africa 032: Product of lunar volcanism. *Meteorit. Planet. Sci.* **37**, 371–394 (2002).
16. Joy, K. H., Crawford, I. A., Russell, S. S. & Kearsley, A. T. LAP 02205, LAP 02224 and LAP 02226 Lunar Mare Basaltic Meteorites. Part 1: Petrography and Mineral Chemistry. In *36th Annual Lunar and Planetary Science Conference* (eds. Mackwell, S. & Stansbery, E.) 1697 (2005).
17. Day, J. M. D. et al. Comparative petrology, geochemistry, and petrogenesis of evolved, low-Ti lunar mare basalt meteorites from the LaPaz Icefield, Antarctica. *Geochim. Cosmochim. Acta* **70**, 1581–1600 (2006).
18. Borg, L. E. et al. Mechanisms for incompatible-element enrichment on the Moon deduced from the lunar basaltic meteorite Northwest Africa 032. *Geochim. Cosmochim. Acta* **73**, 3963–3980 (2009).
19. Elardo, S. M. et al. The origin of young mare basalts inferred from lunar meteorites Northwest Africa 4734, 032, and LaPaz Icefield 02205. *Meteorit. Planet. Sci.* **49**, 261–291 (2014).
20. Grove, T. L. & Vaniman, D. T. Experimental Petrology of Very Low Ti Basalts and Origin of Luna 24 Ferrobasalt. In *LPI Contributions* (eds. Merrill, R. B. & Papike, J. J.) vol. 304 68 (1977).
21. Coish, R. A. & Taylor, L. A. Mineralogy and petrology of basaltic fragments from the Luna 24 drill core. In Mare Crisium: The view from Luna 24 (eds. Merrill, R. B. & Papike, J. J.) 403–417 (1978).
22. Ryder, G. & Marvin, U. B. On the origin of Luna 24 basalts and soils. In Mare Crisium: The view from Luna 24 (eds. Merrill, R. B. & Papike, J. J.) 339–355 (1978).
23. Hallis, L. J., Anand, M. & Strekopytov, S. Trace-element modelling of mare basalt parental melts: Implications for a heterogeneous lunar mantle. *Geochim. Cosmochim. Acta* **134**, 289–316 (2014).
24. Warren, P. H. & Wasson, J. T. The origin of KREEP. *Rev. Geophys.* **17**, 73–88 (1979).
25. Delano, J. W. Apollo 15 Yellow Glasses: Chemistry and Possible Origins. In Lunar and

Planetary Science Conference 213–215 (1980).

26. Longhi, J. Experimental petrology and petrogenesis of mare volcanics. *Geochim. Cosmochim. Acta* **56**, 2235–2251 (1992).
27. Snyder, G. A., Taylor, L. A. & Neal, C. R. A chemical model for generating the sources of mare basalts: Combined equilibrium and fractional crystallization of the lunar magmasphere. *Geochim. Cosmochim. Acta* **56**, 3809–3823 (1992).
28. Elardo, S. M., Draper, D. S. & Shearer, C. K. Lunar Magma Ocean crystallization revisited: Bulk composition, early cumulate mineralogy, and the source regions of the highlands Mg-suite. *Geochim. Cosmochim. Acta* **75**, 3024–3045 (2011).
29. Hughes, S. S., Delano, J. W. & Schmitt, R. A. Petrogenetic modeling of 74220 high-Ti orange volcanic glasses and the Apollo 11 and 17 high-Ti mare basalts. In *Lunar Planet. Sci. Conf. Proc.* **19**, 175–188 (1989).
30. Rapp, J. F. & Draper, D. S. Fractional crystallization of the lunar magma ocean: Updating the dominant paradigm. *Meteorit. Planet. Sci.* **53**, 1432–1455 (2018).
31. Johnson, T. E., Morrissey, L. J., Nemchin, A. A., Gardiner, N. J. & Snape, J. F. The phases of the Moon: Modelling crystallisation of the lunar magma ocean through equilibrium thermodynamics. *Earth Planet. Sci. Lett.* **556**, 116721 (2021).
32. Neal, C. R. & Taylor, L. A. Petrogenesis of mare basalts: A record of lunar volcanism. *Geochim. Cosmochim. Acta* **56**, 2177–2211 (1992).
33. Zeigler, R. A., Korotev, R. L., Jolliff, B. L. & Haskin, L. A. Petrography and geochemistry of the LaPaz Icefield basaltic lunar meteorite and source crater pairing with Northwest Africa 032. *Meteorit. Planet. Sci.* **40**, 1073–1101 (2005).
34. Neal, C. R. et al. Basalt generation at the Apollo 12 site, Part 1: New data, classification, and re-evaluation. Meteoritics 29, 334–348 (1994).
35. Schnare, D. W., Day, J. M. D., Norman, M. D., Liu, Y. & Taylor, L. A. A laser-ablation ICP-MS study of Apollo 15 low-titanium olivine-normative and quartz-normative mare basalts. Geochim. Cosmochim. Acta 72, 2556–2572 (2008).
36. Baldridge, W. S., Beaty, D. W., Hill, S. M. R. & Albee, A. L. The petrology of the Apollo 12 pigeonite basalt suite. In Lunar and Planetary Science Conference Proceedings vol. 10 141–179 (1979).
37. McKay, G. A. Crystal/liquid partitioning of REE in basaltic systems: Extreme fractionation of REE in olivine. *Geochim. Cosmochim. Acta* **50**, 69–79 (1986).
38. Yao, L., Sun, C. & Liang, Y. A parameterized model for REE distribution between low-Ca pyroxene and basaltic melts with applications to REE partitioning in low-Ca pyroxene

along a mantle adiabat and during pyroxenite-derived melt and peridotite interaction. *Contrib. to Mineral. Petrol.* **164**, 261–280 (2012).

39. McKay, G., Le, L. & Wagstaff, J. Constraints on the Origin of the Mare Basalt Europium Anomaly: REE Partition Coefficients for Pigeonite. In *Lunar and Planetary Science Conference* vol. 22 883 (1991).
40. Phinney, W. C. & Morrison, D. A. Partition coefficients for calcic plagioclase: Implications for Archean anorthosites. *Geochim. Cosmochim. Acta* **54**, 1639–1654 (1990).
41. Nyquist, L. E., Wooden, J. L., Shih, C.-Y., Wiesmann, H. & Bansal, B. M. Isotopic and REE studies of lunar basalt 12038: Implications for petrogenesis of aluminous mare basalts. Earth Planet. Sci. Lett. 55, 335–355 (1981).
42. Dickinson, T. et al. Apollo 14 aluminous mare basalts and their possible relationship to KREEP. J. Geophys. Res. Solid Earth 90, C365–C374 (1985).
43. Elkins-Tanton, L. T., Burgess, S. & Yin, Q.-Z. The lunar magma ocean: Reconciling the solidification process with lunar petrology and geochronology. *Earth Planet. Sci. Lett.* **304**, 326–336 (2011).
44. Longhi, J. A new view of lunar ferroan anorthosites: Postmagma ocean petrogenesis. *J. Geophys. Res. Planets* **108**, (2003).
45. Shearer, C. K. et al. Thermal and magmatic evolution of the Moon. *Rev. Mineral. Geochemistry* **60**, 365–518 (2006).
46. Charlier, B., Grove, T. L., Namur, O. & Holtz, F. Crystallization of the lunar magma ocean and the primordial mantle-crust differentiation of the Moon. *Geochim. Cosmochim. Acta* **234**, 50–69 (2018).
47. Snyder, G. A., Taylor, L. A. & Neal, C. R. The sources of mare basalts: A model involving lunar magma ocean crystallization, plagioclase flotation, and trapped instantaneous residual liquid. In Mare Volcanism and Basalt Petrogenesis: Astounding Fundamental Concepts 53 (1991).
48. Melosh, H. J. et al. South Pole–Aitken basin ejecta reveal the Moon's upper mantle. *Geology* **45**, 1063–1066 (2017).
49. Moriarty, D. P., Dygert, N., Valencia, S. N., Watkins, R. N. & Petro, N. E. The search for lunar mantle rocks exposed on the surface of the Moon. *Nat. Commun.* **12**, 1–11 (2021).
50. Ringwood, A.E. and Essene, E., 1970. Petrogenesis of lunar basalts and the internal constitution and origin of the moon. Science, 167(3918), pp.607-610.
51. Elardo, S. M., Shearer, C. K., Vander Kaaden, K. E., McCubbin, F. M. & Bell, A. S. Petrogenesis of primitive and evolved basalts in a cooling Moon: Experimental constraints

from the youngest known lunar magmas. *Earth Planet. Sci. Lett.* **422**, 126–137 (2015).

52. Kesson, S. E. Mare basalts: melting experiments and petrogenetic interpretations. Lunar Planet. Sci. Conf. Proc. 1, 921–944 (1975).
53. Walker, D., Longhi, J., Stolper, E. M., Grove, T. L. & Hays, J. F. Slowly Cooled Microgabbros 15065 and 15555. In Lunar and Planetary Science Conference vol. 8 (1977).
54. Elardo, S. M. & Astudillo Manoslava, D. F. Ancient igneous differentiation trends in the Moon's crust can be produced by secondary magmatism from a common source. In *52nd Lunar and Planetary Science Conference* 2313 (2021).
55. Astudillo Manosalva, D. F. & Elardo, S. M. The Accuracy of Perple_X, pMelts, and MAGPOX in Modelling Equilibrium Crystallization of Lunar and Martian Basalt Compositions and Their Multiple Saturation Points. In LPI Contributions vol. 2678 2343 (2022).
56. Sobolev, A.V., Hofmann, A.W., Sobolev, S.V. and Nikogosian, I.K., 2005. An olivine-free mantle source of Hawaiian shield basalts. Nature, 434(7033), pp.590-597.
57. Sobolev, A.V., Hofmann, A.W., Kuzmin, D.V., Yaxley, G.M., Arndt, N.T., Chung, S.L., Danyushevsky, L.V., Elliott, T., Frey, F.A., Garcia, M.O. and Gurenko, A.A., 2007. The amount of recycled crust in sources of mantle-derived melts. science, 316(5823), pp.412-417.
58. Herzberg, C., 2011. Identification of source lithology in the Hawaiian and Canary Islands: Implications for origins. Journal of Petrology, 52(1), pp.113-146.
59. Yang, Z.F. and Zhou, J.H., 2013. Can we identify source lithology of basalt?. Scientific Reports, 3(1), pp.1-7.
60. Yang, Z.F., Li, J., Jiang, Q.B., Xu, F., Guo, S.Y., Li, Y. and Zhang, J., 2019. Using major element logratios to recognize compositional patterns of basalt: Implications for source lithological and compositional heterogeneities. Journal of Geophysical Research: Solid Earth, 124(4), pp.3458-3490.
61. Day, J.M.D., 2020. Metal grains in lunar rocks as indicators of igneous and impact processes. Meteoritics & Planetary Science, 55(8).
62. Warren, P. H. & Kallemeyn, G. W. Geochemical Investigation of two lunar meteorites: Y-793197 and Asuka-881757. Antarct. Meteor. Res. 6, 35 (1993).
63. Koeberl, C., Kurat, G. & Brandstätter, F. Gabbroic lunar mare meteorites Asuka-881757 (Asuka-31) and Yamato-793169: Geochemical and mineralogical study. Antarct. Meteor. Res. 6, 14 (1993).
64. Valencia, S. N., Jolliff, B. L. & Korotev, R. L. Petrography, relationships, and petrogenesis

of the gabbroic lithologies in Northwest Africa 773 clan members Northwest Africa 773, 2727, 3160, 3170, 7007, and 10656. Meteorit. Planet. Sci. 54, 2083–2115 (2019).

65. Fagan, T. J., Kashima, D., Wakabayashi, Y. & Suginohara, A. Case study of magmatic differentiation trends on the Moon based on lunar meteorite Northwest Africa 773 and comparison with Apollo 15 quartz monzodiorite. *Geochim. Cosmochim. Acta* **133**, 97–127 (2014).
66. Joy, K. H., Crawford, I. A. & Russell, S. S. The Petrography and Geochemistry of Lunar Meteorite Regolith Breccia MET 01210. Meteorit. Planet. Sci. Suppl. 41, 5221 (2006).
67. Nyquist, L. E. Lunar Rb-Sr chronology. *Phys. Chem. Earth* **10**, 103–142 (1977).
68. Nyquist, L. E. et al. Rb-Sr systematics for chemically defined Apollo 15 and 16 materials. Lunar Planet. Sci. Conf. Proc. 4, 1823 (1973).
69. Nyquist, L. E. & Shih, C. Y. The isotopic record of lunar volcanism. Geochim. Cosmochim. Acta 56, 2213–2234 (1992).
70. Nyquist, L. E. et al. 146Sm-142Nd formation interval for the lunar mantle. Geochim. Cosmochim. Acta 59, 2817–2837 (1995).
71. Putirka, K. D. Thermometers and Barometers for Volcanic Systems. Rev. Mineral. Geochemistry 69, 61–120 (2008).
72. Yang, Z.F., Li, J., Liang, W.F. and Luo, Z.H., 2016. On the chemical markers of pyroxenite contributions in continental basalts in Eastern China: Implications for source lithology and the origin of basalts. Earth-Science Reviews, 157, pp.18-31.
73. Nekvasil, H. The Origin and Evolution of Silica-saturated Alkalic Suites: an Experimental Study. J. Petrol. 45, 693–721 (2004).
74. Warren, P. H. & Taylor, G. J. The Moon. Treatise on Geochemistry: Second Edition vol. 2 (2013).
75. Elkins-Tanton, L. T., Hager, B. H. & Grove, T. L. Magmatic effects of the lunar late heavy bombardment. Earth Planet. Sci. Lett. 222, 17–27 (2004).
76. Beattie, P. Olivine-melt and orthopyroxene-melt equilibria. Contrib. to Mineral. Petrol. 115, 103–111 (1993).
77. Wang, X. et al. A new clinopyroxene thermobarometer for mafic to intermediate magmatic systems. Eur. J. Mineral. 33, 621–637 (2021).
78. Mare Basalt Database by C. Neal (http://www.nd.edu/~cneal/Lunar-L/).
