# Supplementary material for: A changing thermal regime revealed from shallow to deep basalt source melting in the Moon
Source: Nat Commun. 2022 Dec 9;13:7594. doi: 10.1038/s41467-022-35260-y (PMC9734159; doi:10.1038/s41467-022-35260-y)
Supplement: Supplementary file 3 — Description of Additional Supplementary Files [file 41467_2022_35260_MOESM3_ESM.pdf]

Description of Additional Supplementary Files:

Supplementary Data 1| Representative electron microprobe analyses of minerals in A881757.

Supplementary Data 2|Bulk rock chemistry of A-881757, MIL 05035, Y-793169, MET 01210, and other similar rock types.

Supplementary Data 3| Estimated trace element compositions of the mantle sources (average of 75-80 PCS) of YAMM basalts, Kalahari 009, NWA 032, LAP basalts, and Luna 24 ferrobasalts. The assumed source mineralogy for each meteorite and melting model are described in Supplementary Text 3 and in Methods, respectively.

Supplementary Data 4| Estimated formation P-T, mantle potential temperature and heat flow for A-881757 and other non-KREEP basalts.
